# Supplementary material for: Light-enhanced molecular polarity enabling multispectral color-cognitive memristor for neuromorphic visual system
Source: Nat Commun. 2023 Sep 18;14:5775. doi: 10.1038/s41467-023-41419-y (PMC10507016; doi:10.1038/s41467-023-41419-y)
Supplement: Supplementary file 1 — Supplementary Information [file 41467_2023_41419_MOESM1_ESM.pdf]

Supplementary Information

Light-enhanced molecular polarity enabling multispectral color-cognitive memristor for neuromorphic visual system

Jongmin Lee[1,2], Bum Ho Jeong[1,2], Eswaran Kamaraj[3], Dohyung Kim[1,2], Hakjun Kim[1,2] Sanghyuk Park[3,*], Hui Joon Park[1,2,4,*]

[1]Department of Organic and Nano Engineering, Hanyang University, Seoul 04763, Republic of Korea

[2]Human-Tech Convergence Program, Hanyang University, Seoul 04763, Republic of Korea

[3]Department of Chemistry, Kongju National University, Kongju 32588, Republic of Korea

[4]Hanyang Institute of Smart Semiconductor, Seoul 04763, Republic of Korea

*Corresponding authors
huijoon@hanyang.ac.kr (H. J. Park) and spark0920@kongju.ac.kr (S. Park)

Keywords: optoelectronic, memristor, synapse, color-discrimination, neuromorphic visual system

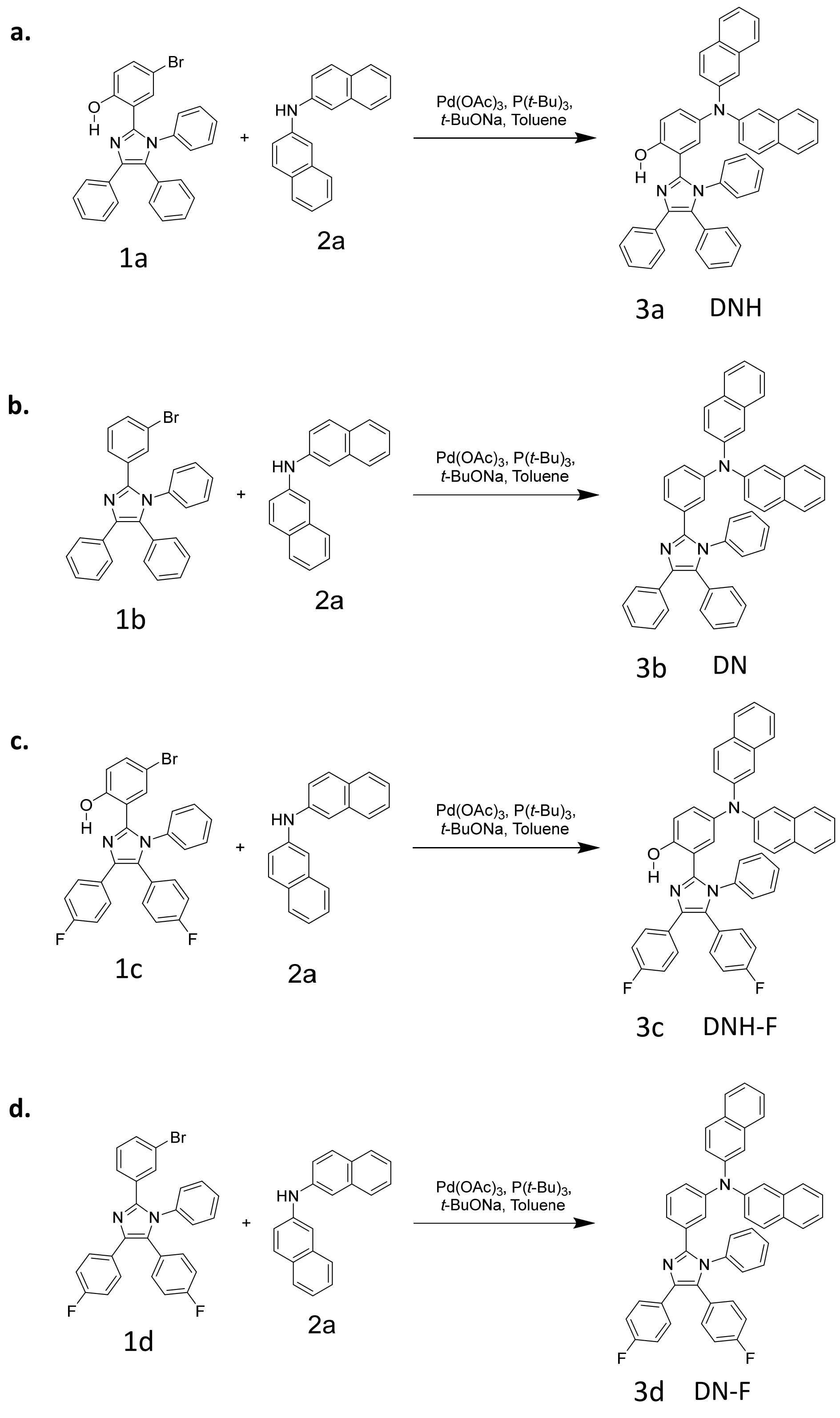

Supplementary Fig. 1 Chemical structures and detailed synthetic routes of UV-responding organic molecules. **a** 4-(di(naphthalen-2-yl)amino)-2-(1,4,5-triphenyl-1H-imidazol-2-yl)phenol (DNH). **b** *N*-(naphthalen-2-yl)-N-(3-(1,4,5-triphenyl-1H-imidazol-2-yl)phenyl)naphthalen-2-amine (DN). **c** 2-(4,5-bis(4-fluorophenyl)-1-phenyl-1H-imidazol-2-yl)-4-(di(naphthalen-2-yl)amino)phenol (DNH-F). **d** *N*-(3-(4,5-bis(4-fluorophenyl)-1-phenyl-1H-imidazol-2-yl)phenyl)-N-(naphthalen-2-yl)naphthalen -2-amine (DN-F).

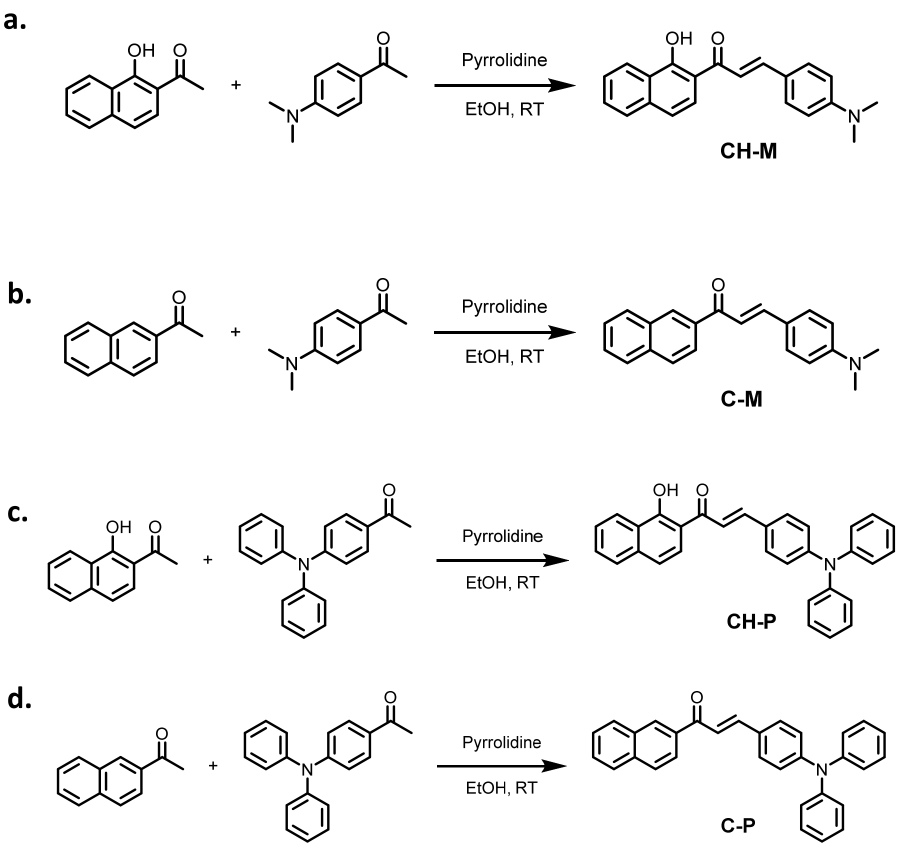

Supplementary Fig. 2 Chemical structures and detailed synthetic routes of visible light-responding organic molecules. **a** (*E*)-3-(4-(dimethylamino)phenyl)-1-(1-hydroxynaphthalen-2-yl)prop-2-en-1-one (CH-M). **b** (*E*)-3-(4-(dimethylamino)phenyl)-1-(naphthalen-2-yl)prop-2-en-1-one (C-M). **c** (*E*)-3-(4-(diphenylamino)phenyl) -1-(1-hydroxynaphthalen-2-yl)prop-2-en-1-one (CH-P). **d** (*E*)-3-(4-(diphenylamino)phenyl)-1-(naphthalen-2-yl)prop-2-en-1-one (C-P).

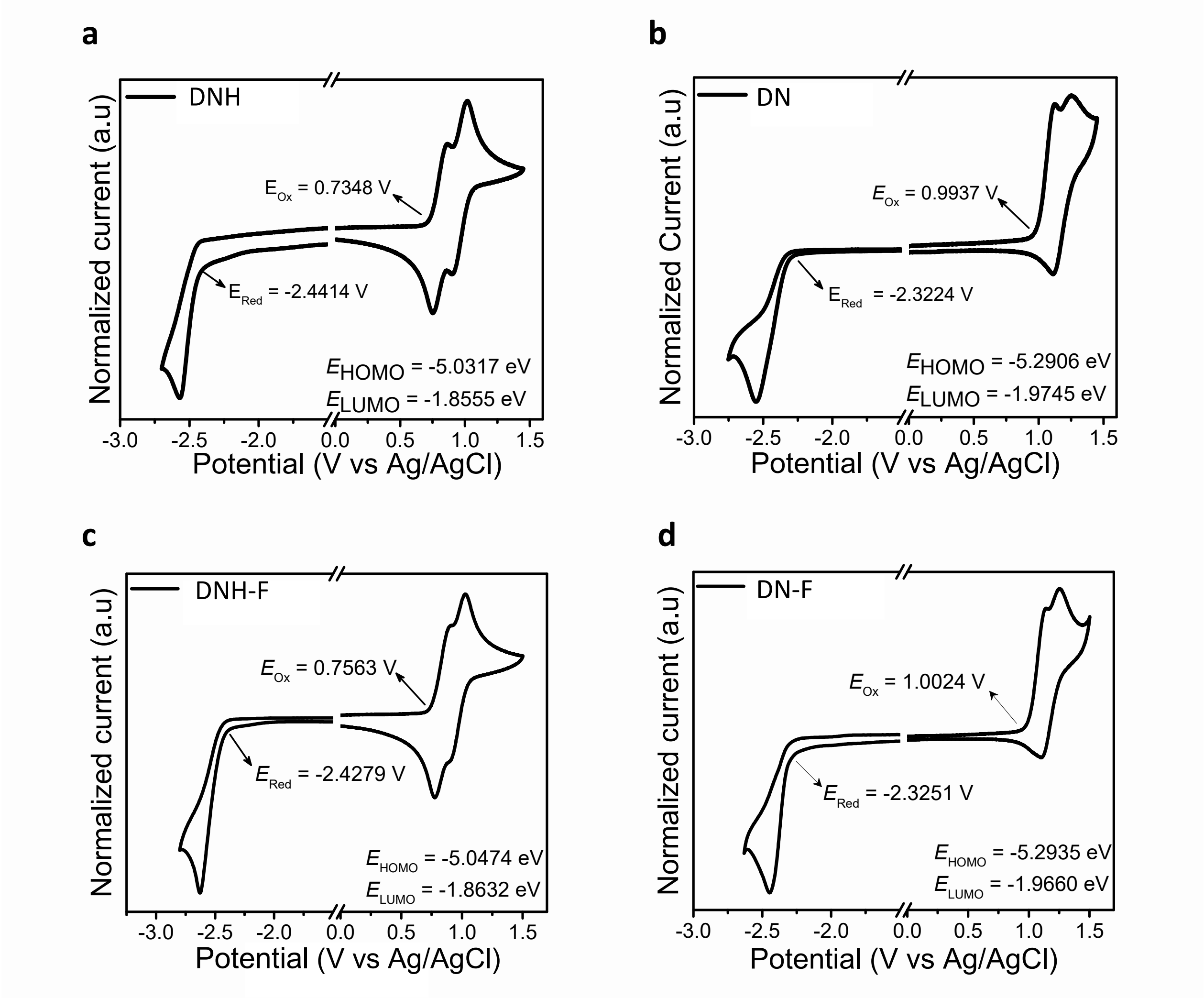

Supplementary Fig. 3 Cyclic voltammetry from 1M TBFPF_6 electrolyte/MC for HOMO and DMF for LUMO. The estimated energy levels of molecules, obtained by cyclic voltammetry results and absorbances, are added to the figures. **a** DNH, **b** DN, **c** DNH-F, and **d** DN-F.

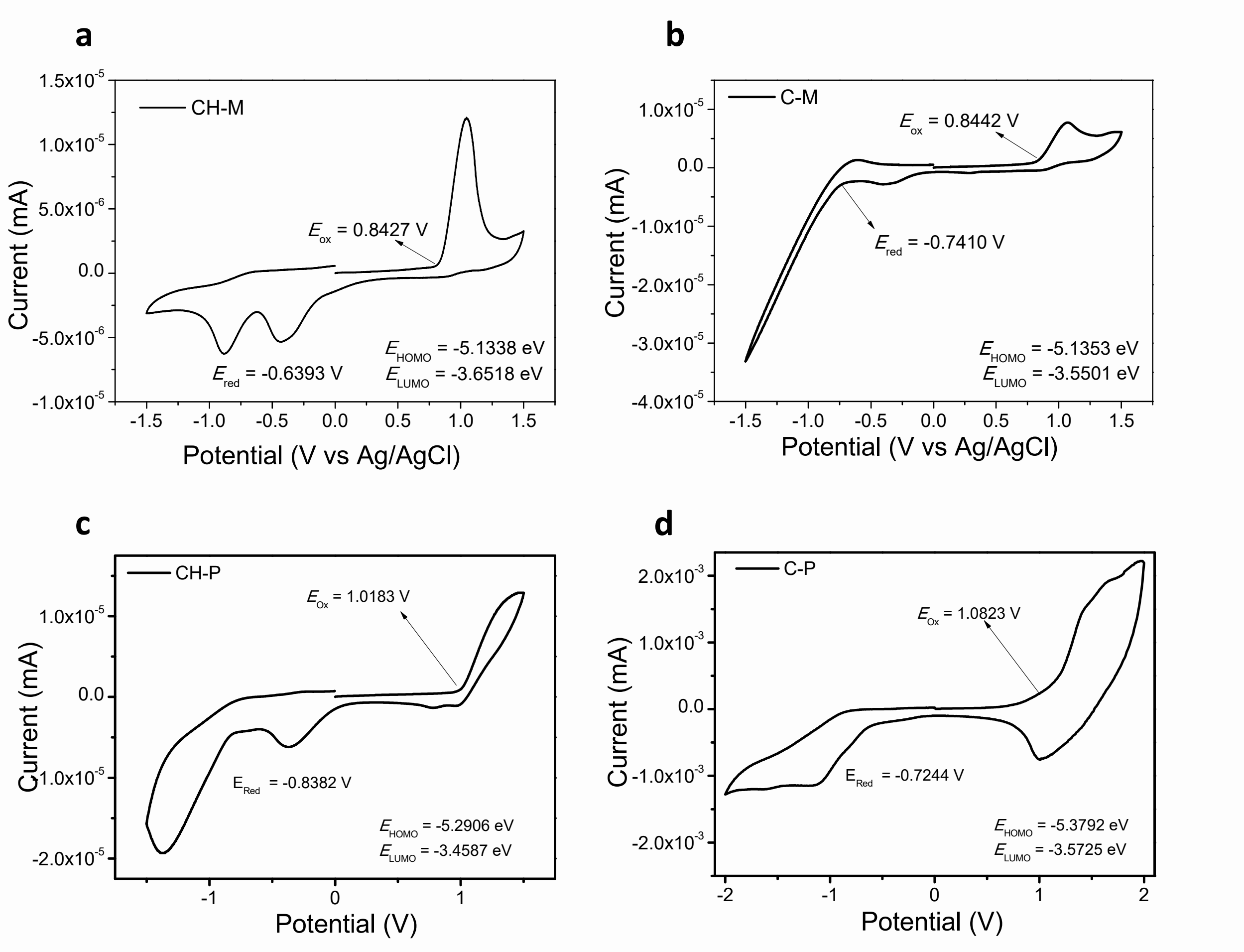

Supplementary Fig. 4 Cyclic voltammetry from 1M $TBFPF_6$ electrolyte/MC for HOMO and DMF for LUMO. The estimated energy levels of molecules, obtained by cyclic voltammetry results and absorbances, are added to the figures. **a** CH-M, **b** C-M, **c** CH-P, and **d** C-P.

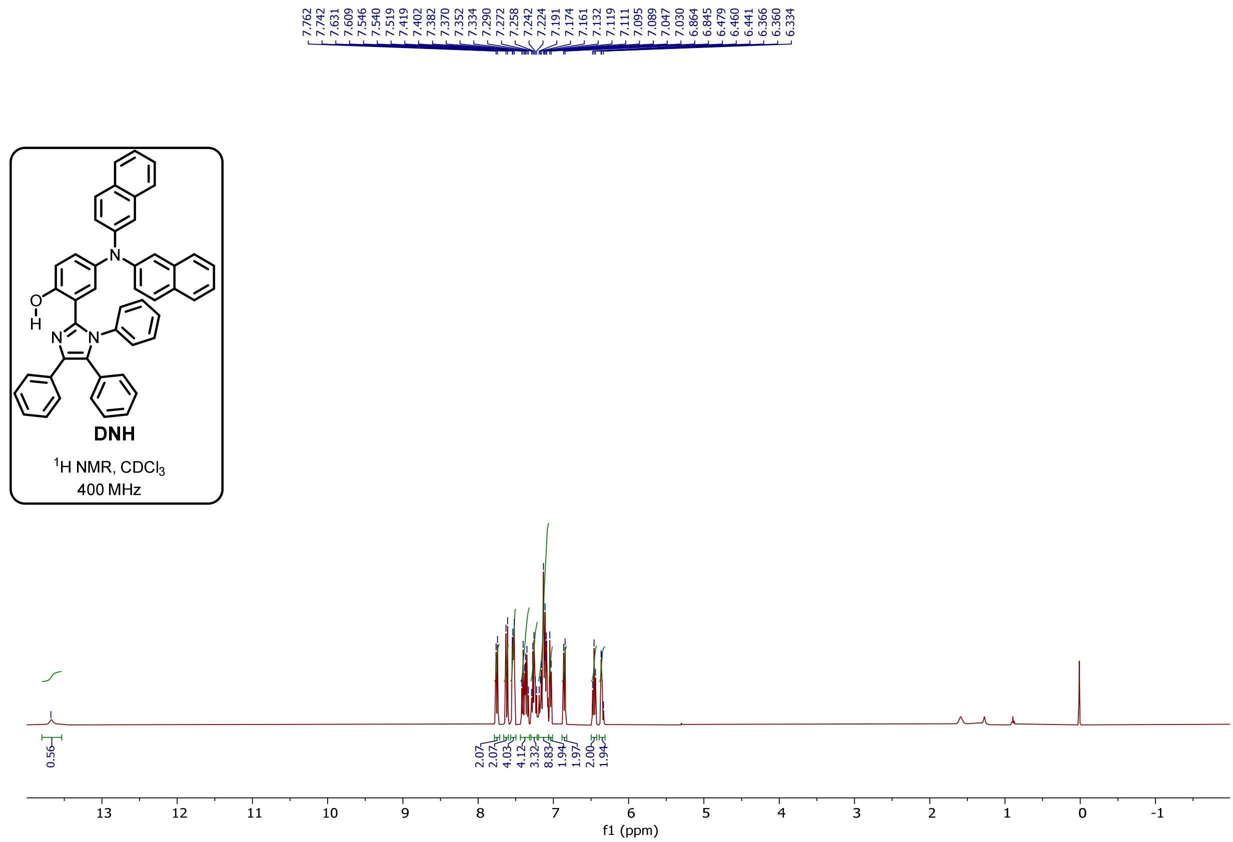

Supplementary Fig. 5 ^{1}H NMR of 4-(di(naphthalen-2-yl)amino)-2-(1,4,5-triphenyl-1H-imidazol-2-yl)phenol (DNH).

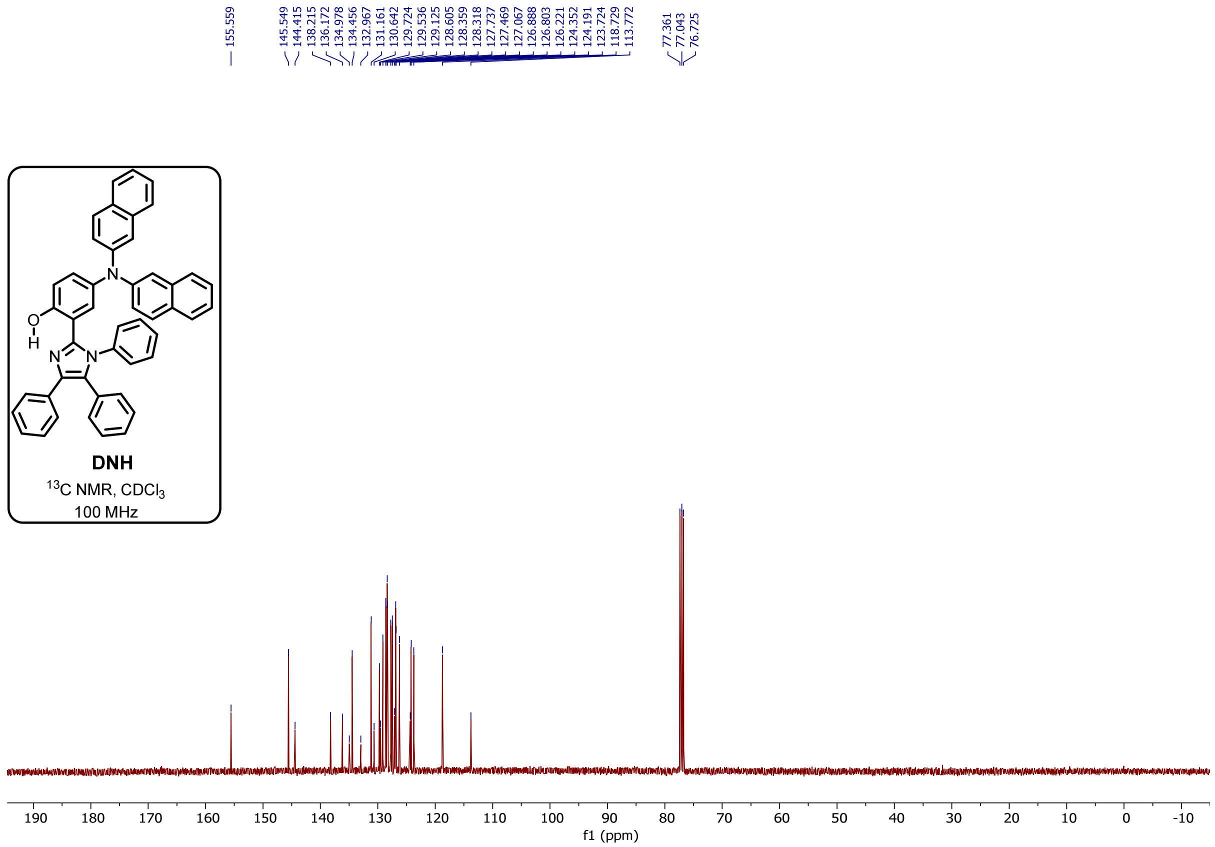

Supplementary Fig. 6 ^{13}C NMR of 4-(di(naphthalen-2-yl)amino)-2-(1,4,5-triphenyl-1H-imidazol-2-yl)phenol (DNH).

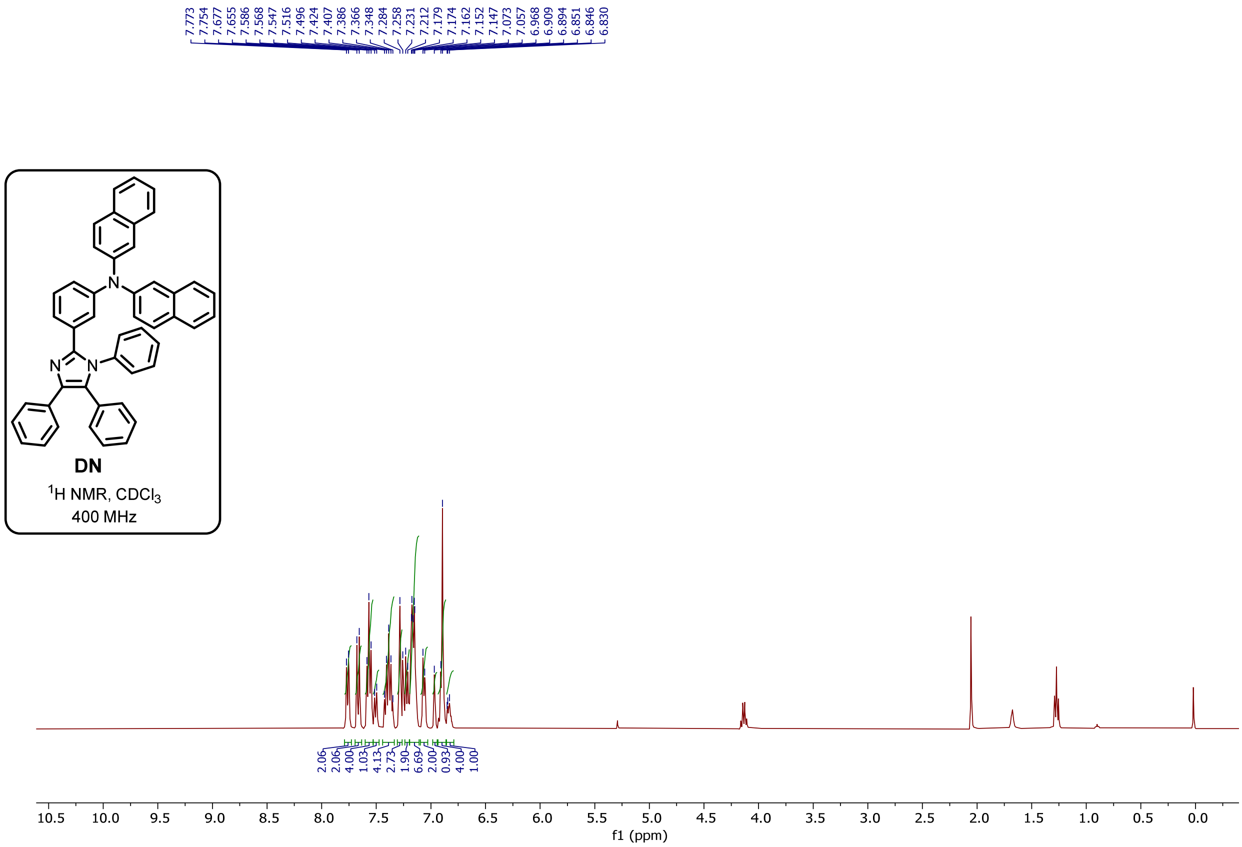

Supplementary Fig. 7 ^{1}H NMR of *N*-(naphthalen-2-yl)-N-(3-(1,4,5-triphenyl-1H-imidazol-2-yl)phenyl)naphthalen-2-amine (DN).

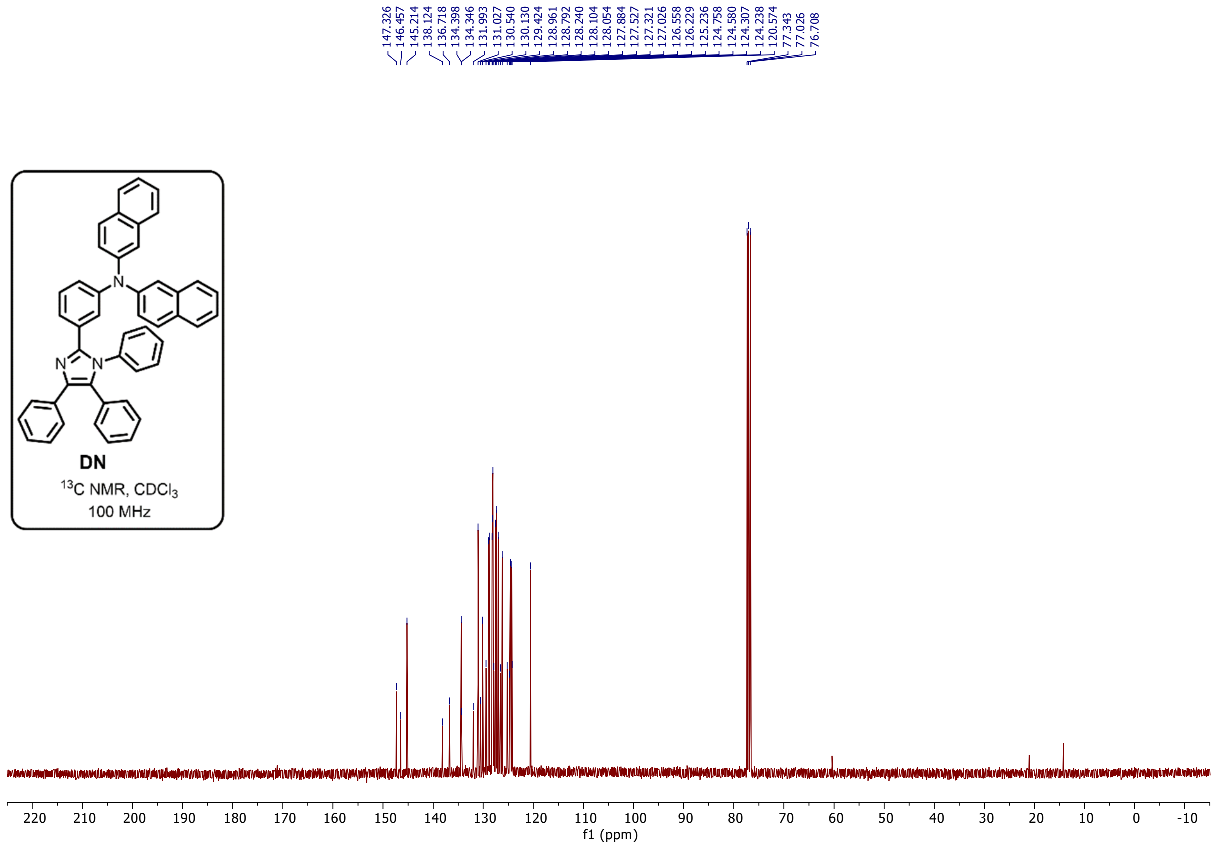

Supplementary Fig. 8 ^{13}C NMR of *N*-(naphthalen-2-yl)-N-(3-(1,4,5-triphenyl-1H-imidazol-2-yl)phenyl)naphthalen-2-amine (DN).

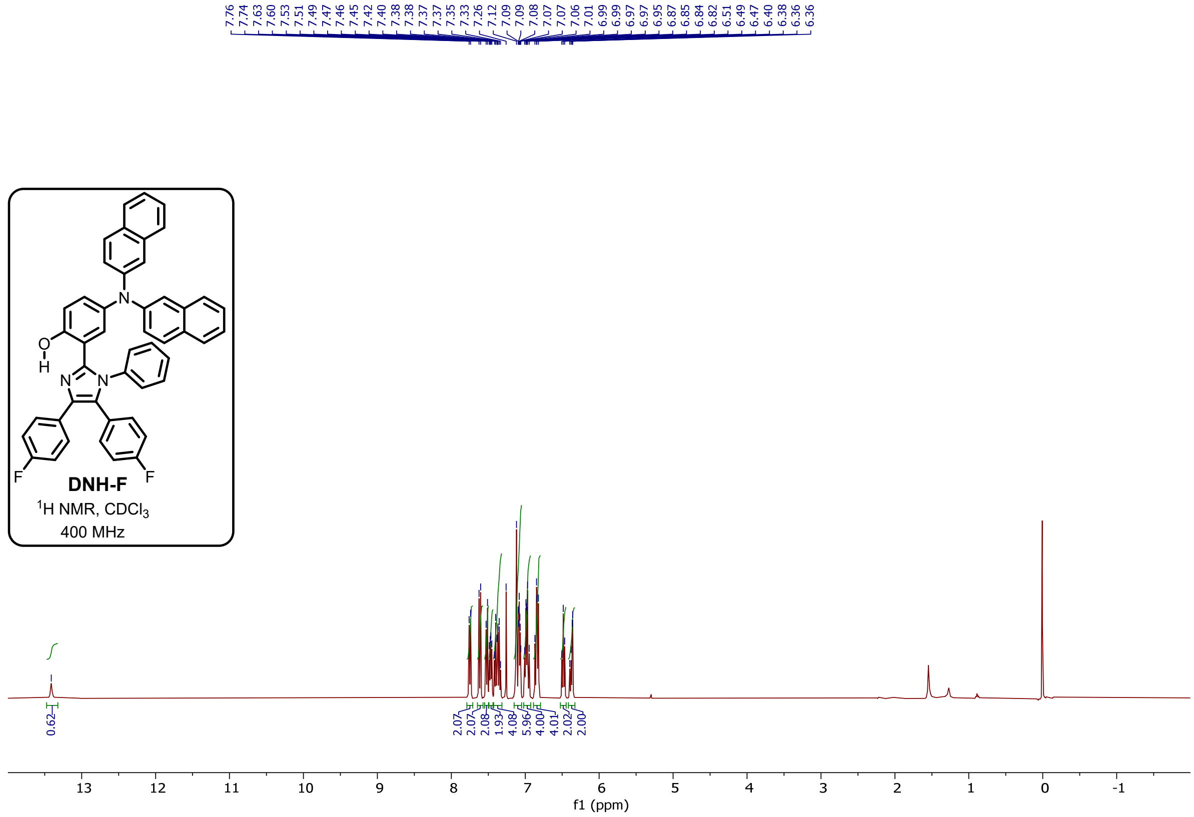

Supplementary Fig. 9 ^{1}H NMR of 2-(4,5-bis(4-fluorophenyl)-1-phenyl-1H-imidazol-2-yl)-4-(di(naphthalen-2-yl)amino)phenol (DNH-F).

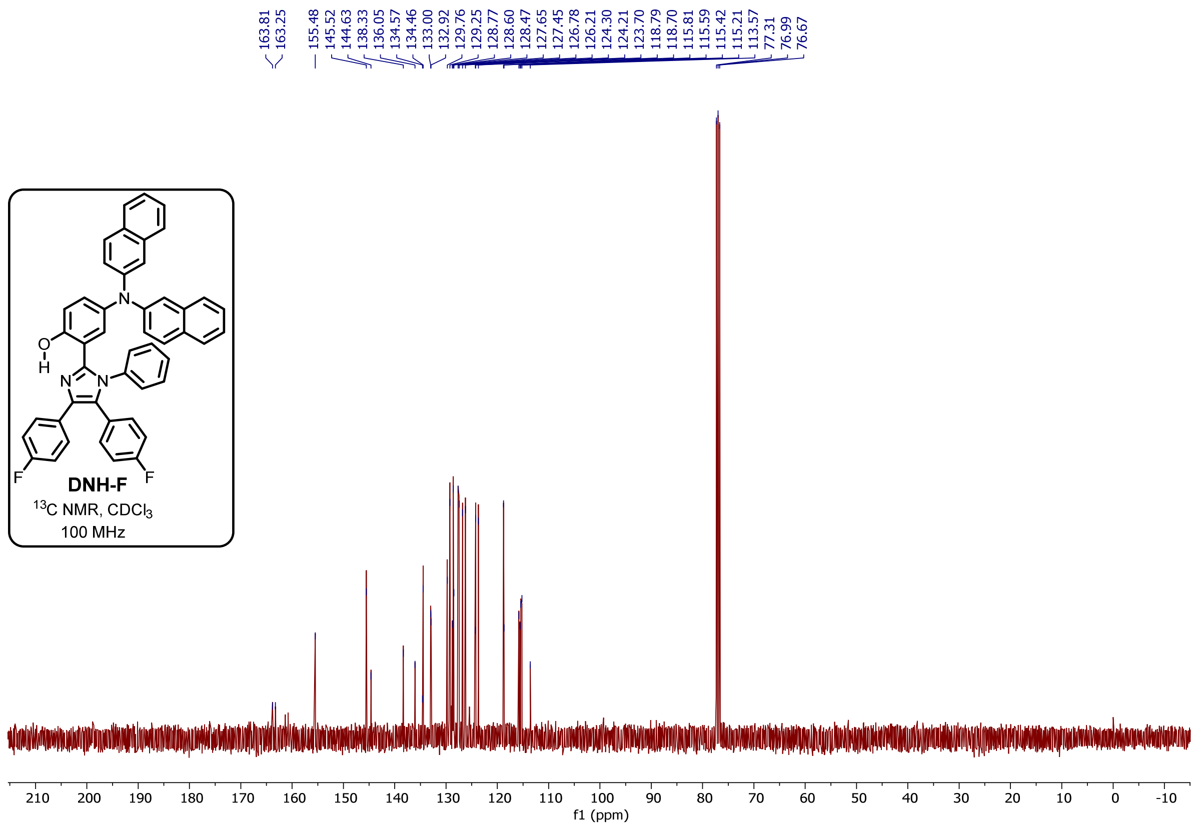

Supplementary Fig. 10 ^{13}C NMR of 2-(4,5-bis(4-fluorophenyl)-1-phenyl-1H-imidazol-2-yl)-4-(di(naphthalen-2-yl)amino)phenol (DNH-F).

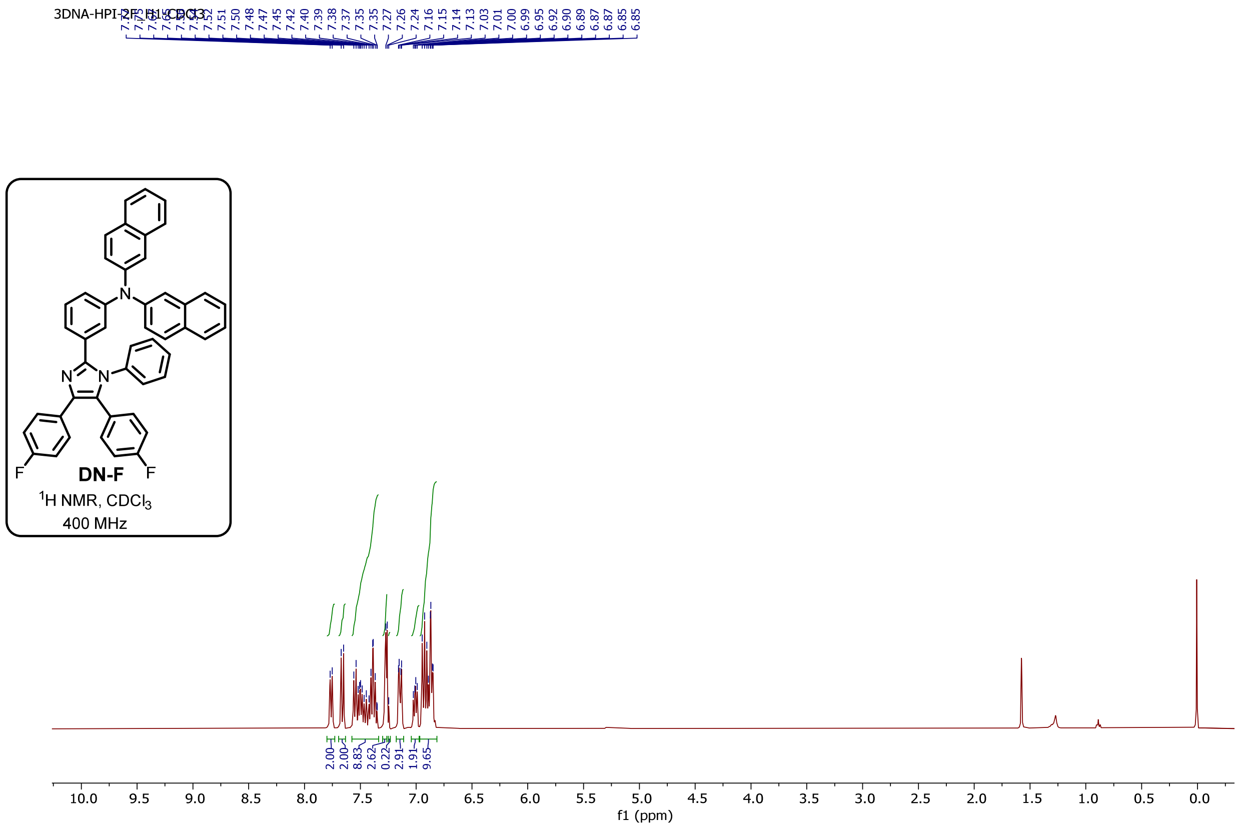

Supplementary Fig. 11 ^{1}H NMR of *N*-(3-(4,5-bis(4-fluorophenyl)-1-phenyl-1H-imidazol-2-yl)phenyl)-N-(naphthalen-2-yl)naphthalen-2-amine (DN-F).

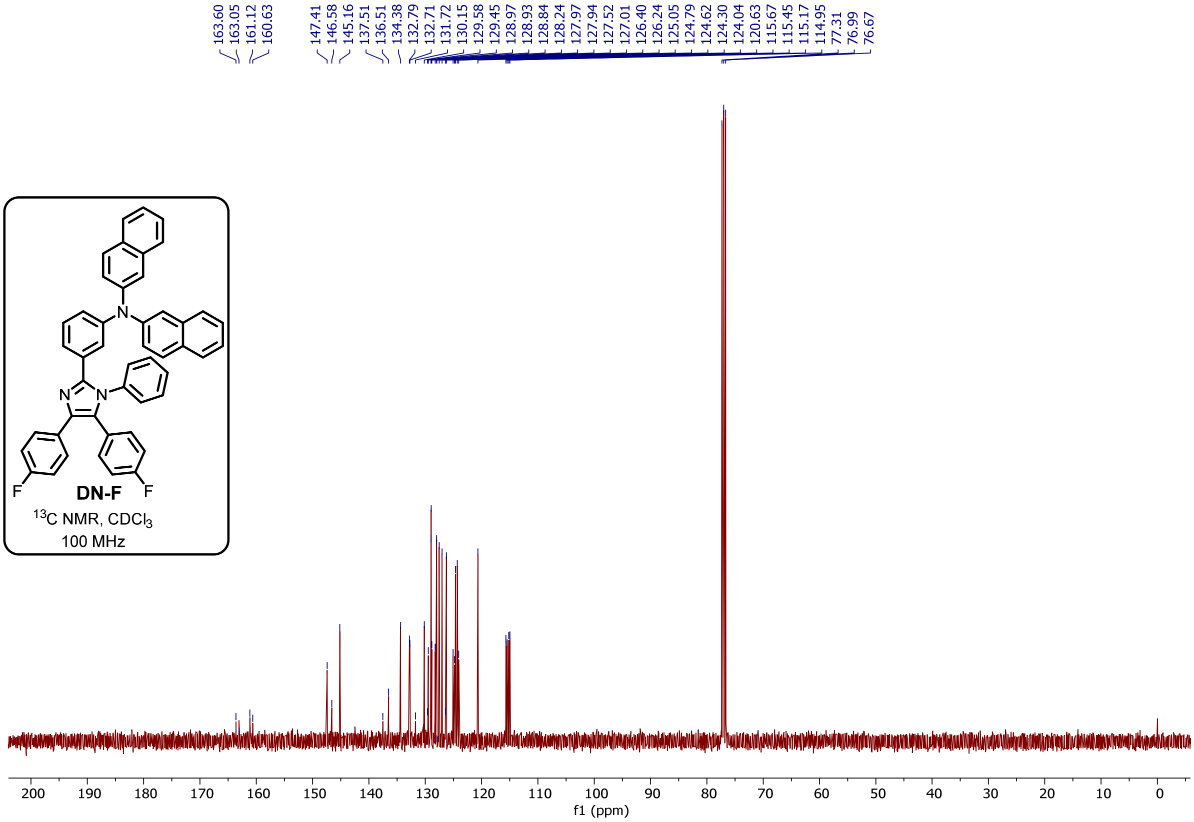

Supplementary Fig. 12 ^{13}C NMR of *N*-(3-(4,5-bis(4-fluorophenyl)-1-phenyl-1H-imidazol-2-yl)phenyl)-N-(naphthalen-2-yl)naphthalen-2-amine (DN-F).

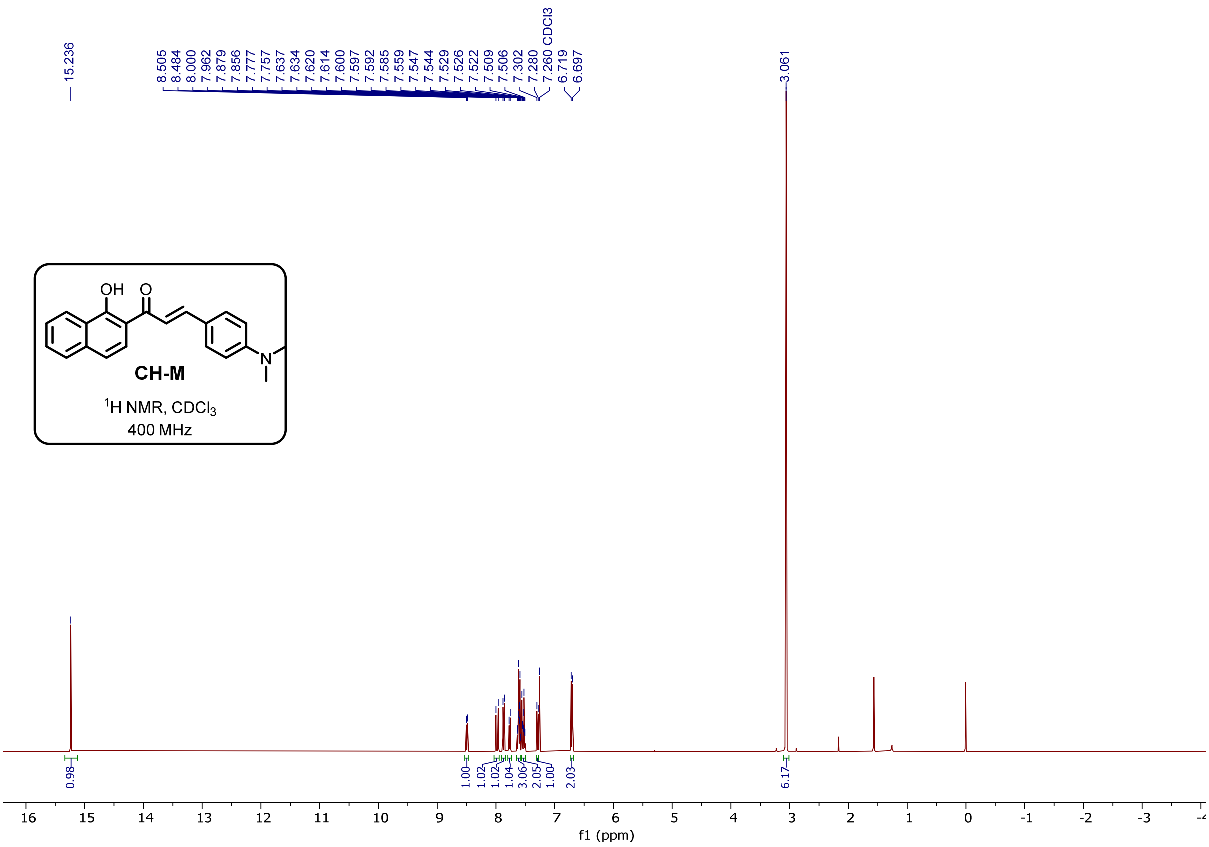

Supplementary Fig. 13 ^{1}H NMR of (*E*)-3-(4-(dimethylamino)phenyl)-1-(1-hydroxynaphthalen-2-yl)prop-2-en-1-one (CH-M).

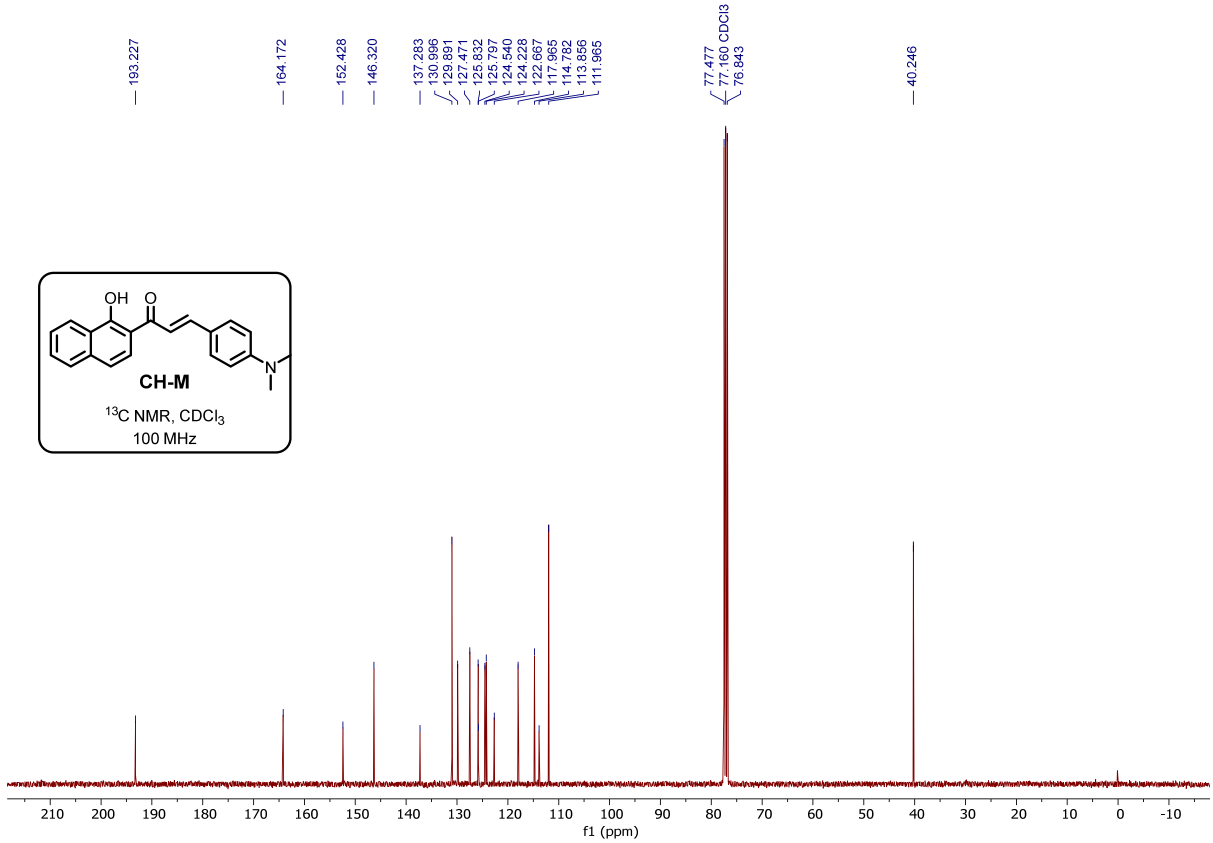

Supplementary Fig. 14 ^{13}C NMR of (*E*)-3-(4-(dimethylamino)phenyl)-1-(1-hydroxynaphthalen-2-yl)prop-2-en-1-one (CH-M).

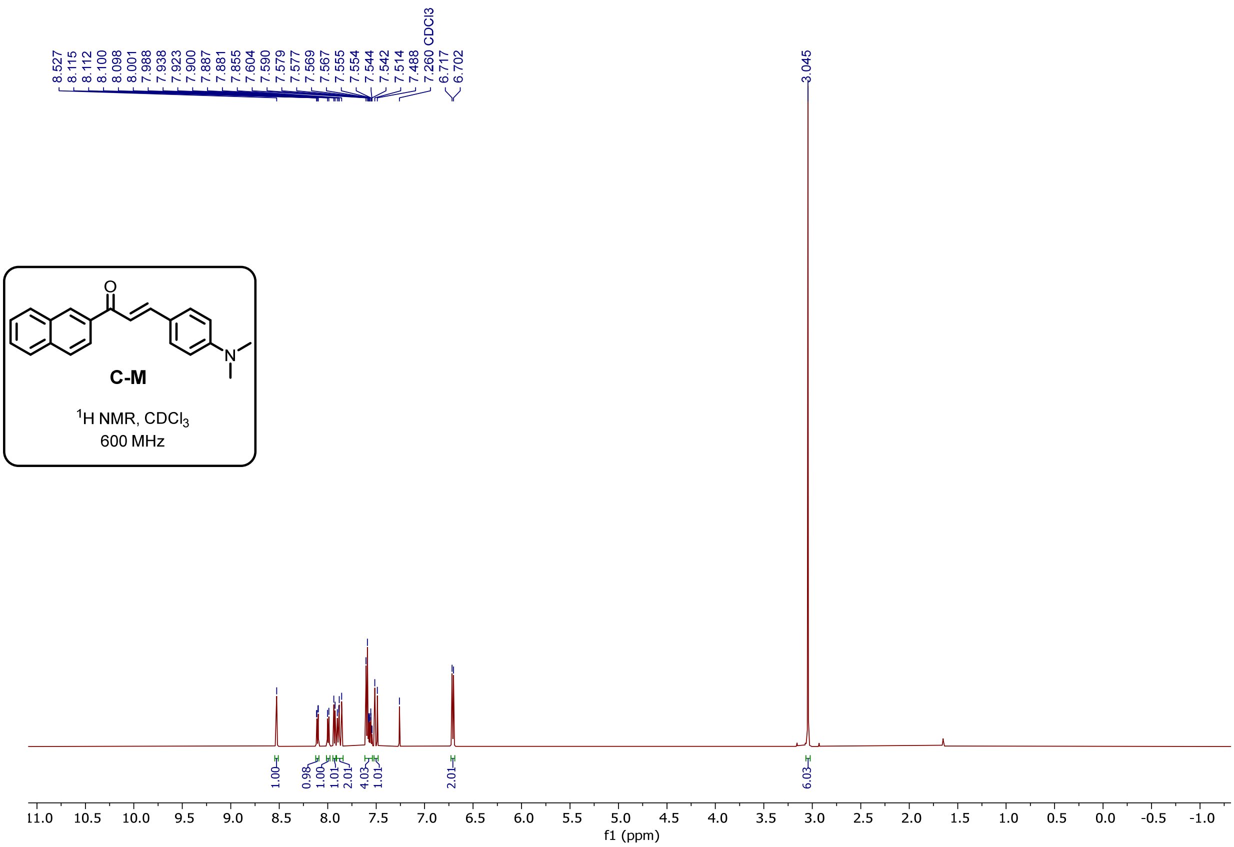

Supplementary Fig. 15 ^{1}H NMR of (*E*)-3-(4-(dimethylamino)phenyl)-1-(naphthalen-2-yl)prop-2-en-1-one (C-M).

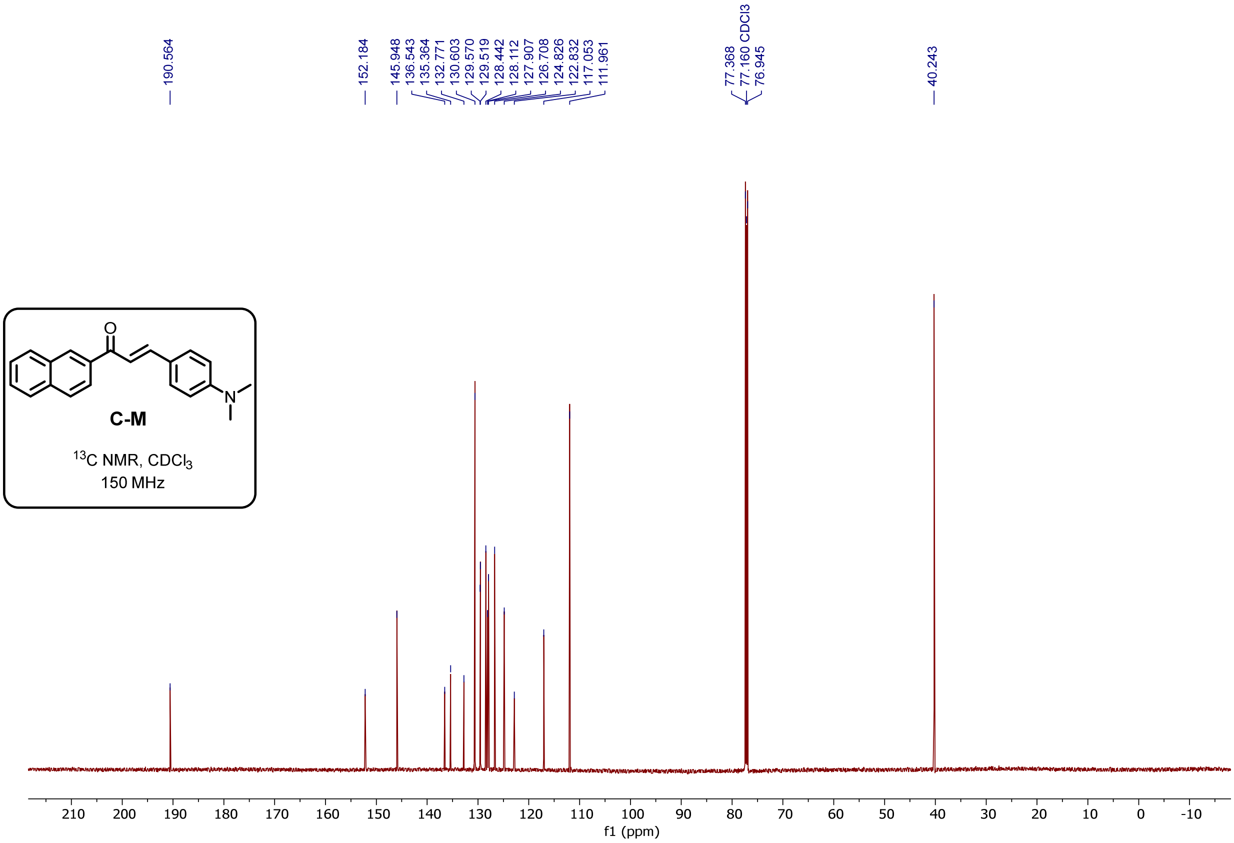

Supplementary Fig. 16 ^{13}C NMR of (*E*)-3-(4-(dimethylamino)phenyl)-1-(naphthalen-2-yl)prop-2-en-1-one (C-M).

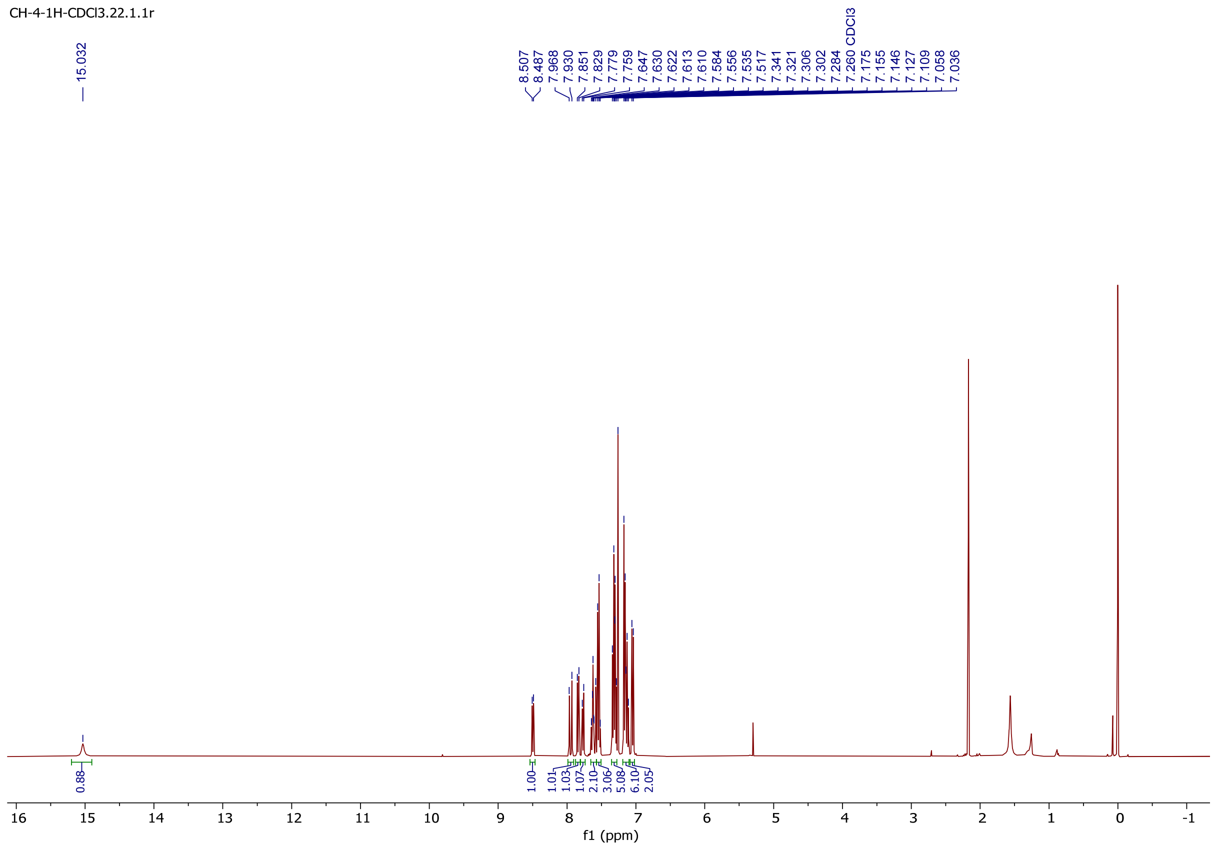

Supplementary Fig. 17 ^{1}H NMR of (*E*)-3-(4-(diphenylamino)phenyl)-1-(1-hydroxynaphthalen-2-yl)prop-2-en-1-one (CH-P).

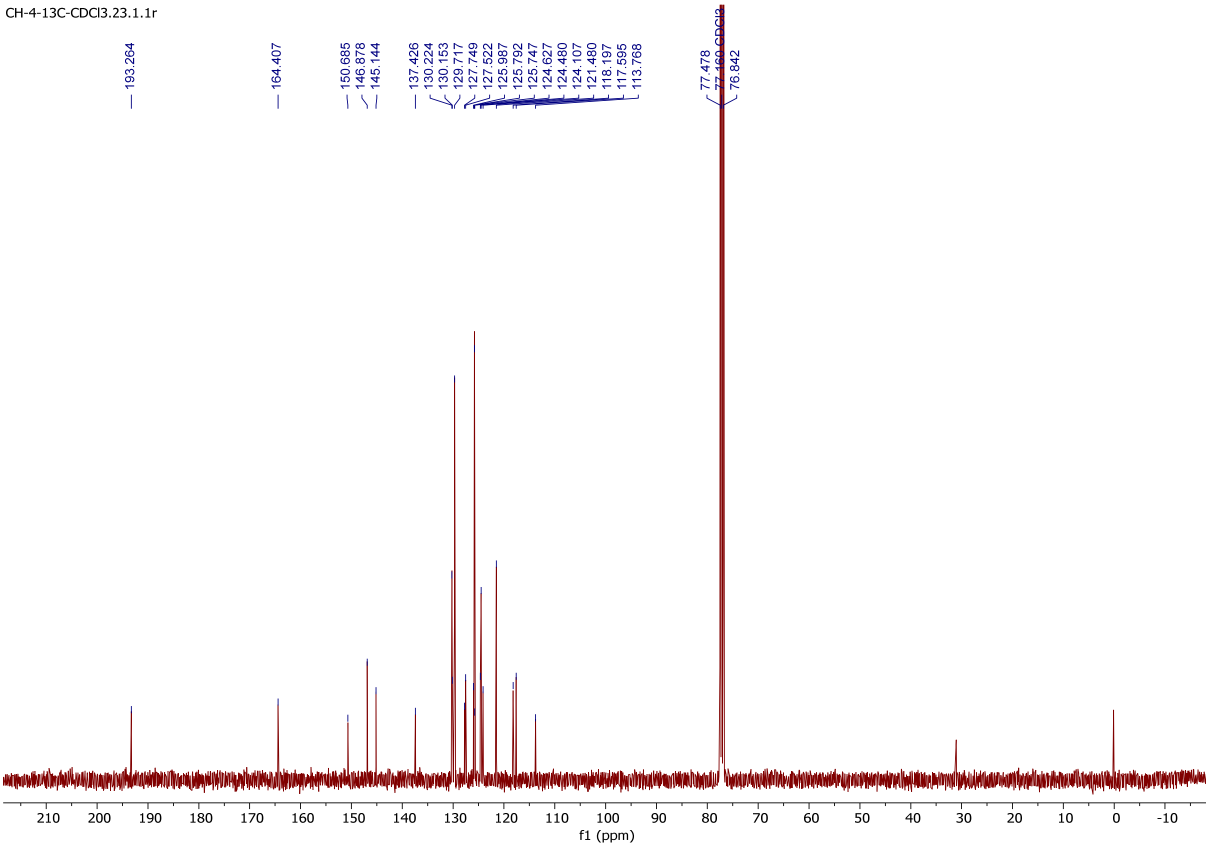

Supplementary Fig. 18 ^{13}C NMR of (*E*)-3-(4-(diphenylamino)phenyl)-1-(1-hydroxynaphthalen-2-yl)prop-2-en-1-one (CH-P).

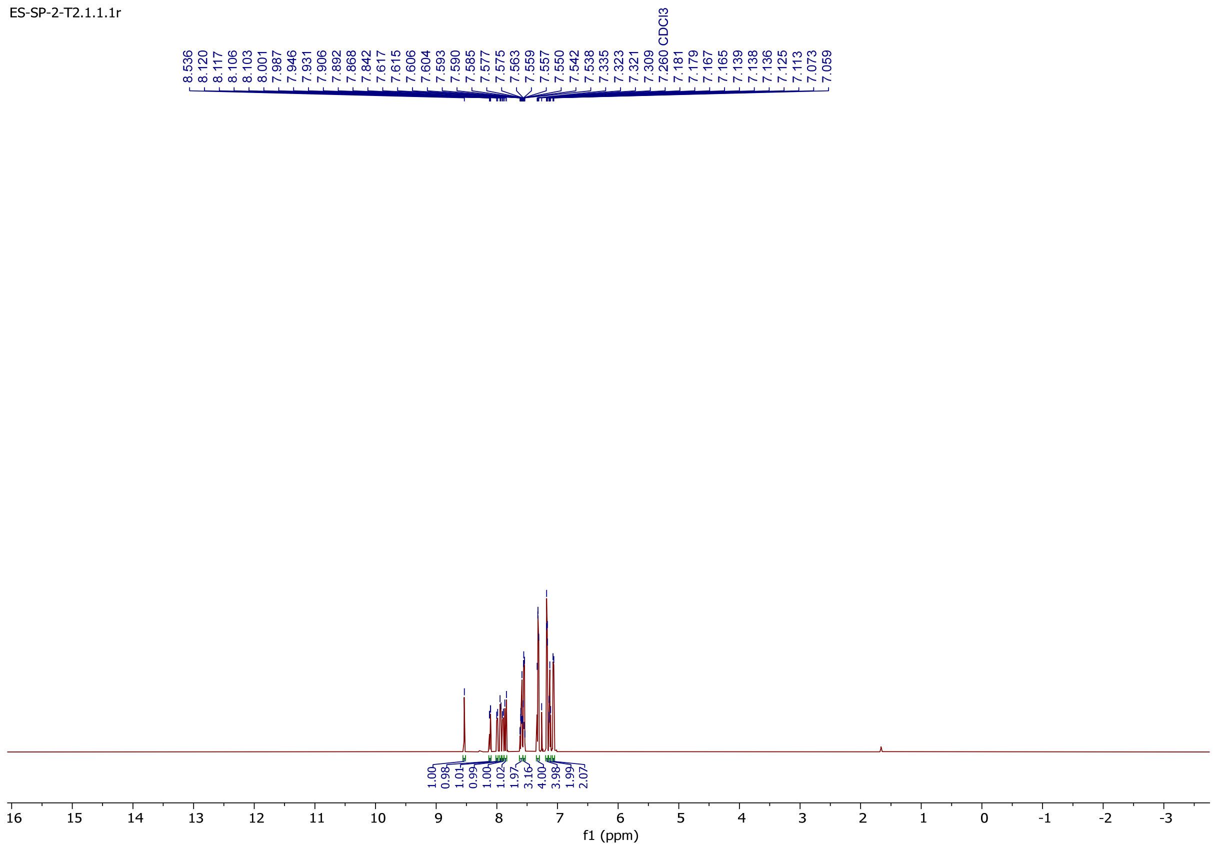

Supplementary Fig. 19 ^{1}H NMR of (*E*)-3-(4-(diphenylamino)phenyl)-1-(naphthalen-2-yl)prop-2-en-1-one (C-P).

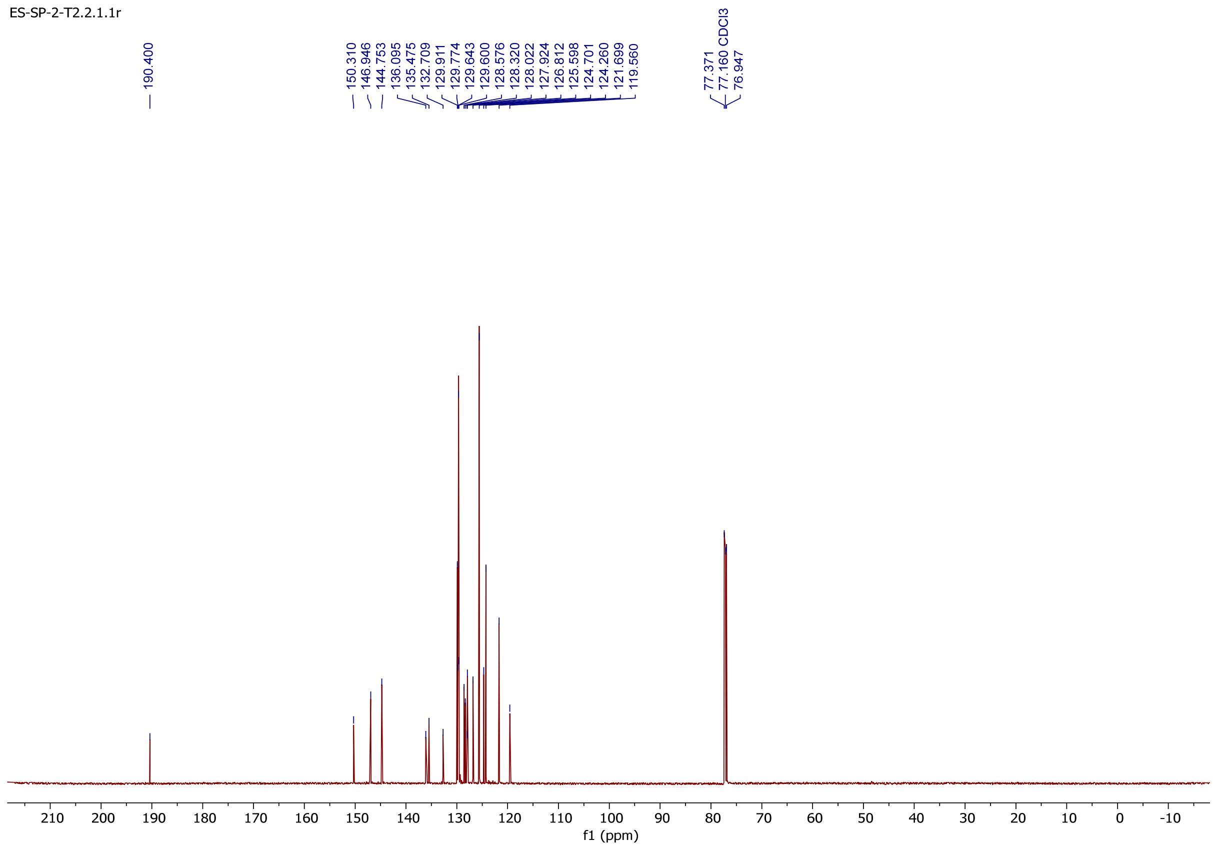

Supplementary Fig. 20 ^{13}C NMR of (*E*)-3-(4-(diphenylamino)phenyl)-1-(naphthalen-2-yl)prop-2-en-1-one (C-P).

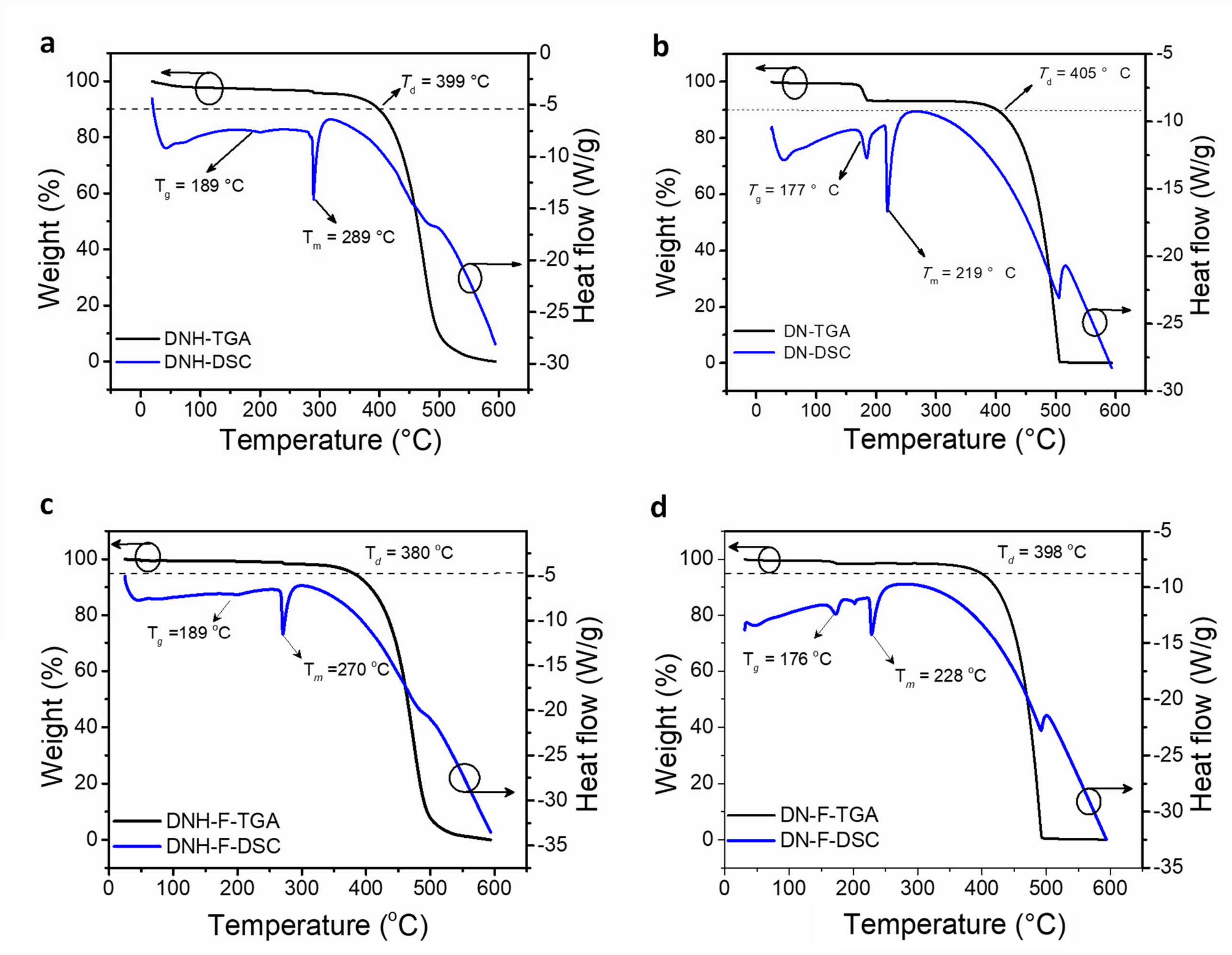

Supplementary Fig. 21 Thermogravimetric analysis (TGA) and differential scanning calorimetry (DSC) results of **a** DHN, **b** DN, **c** DNH-F, and **d** DN-F.

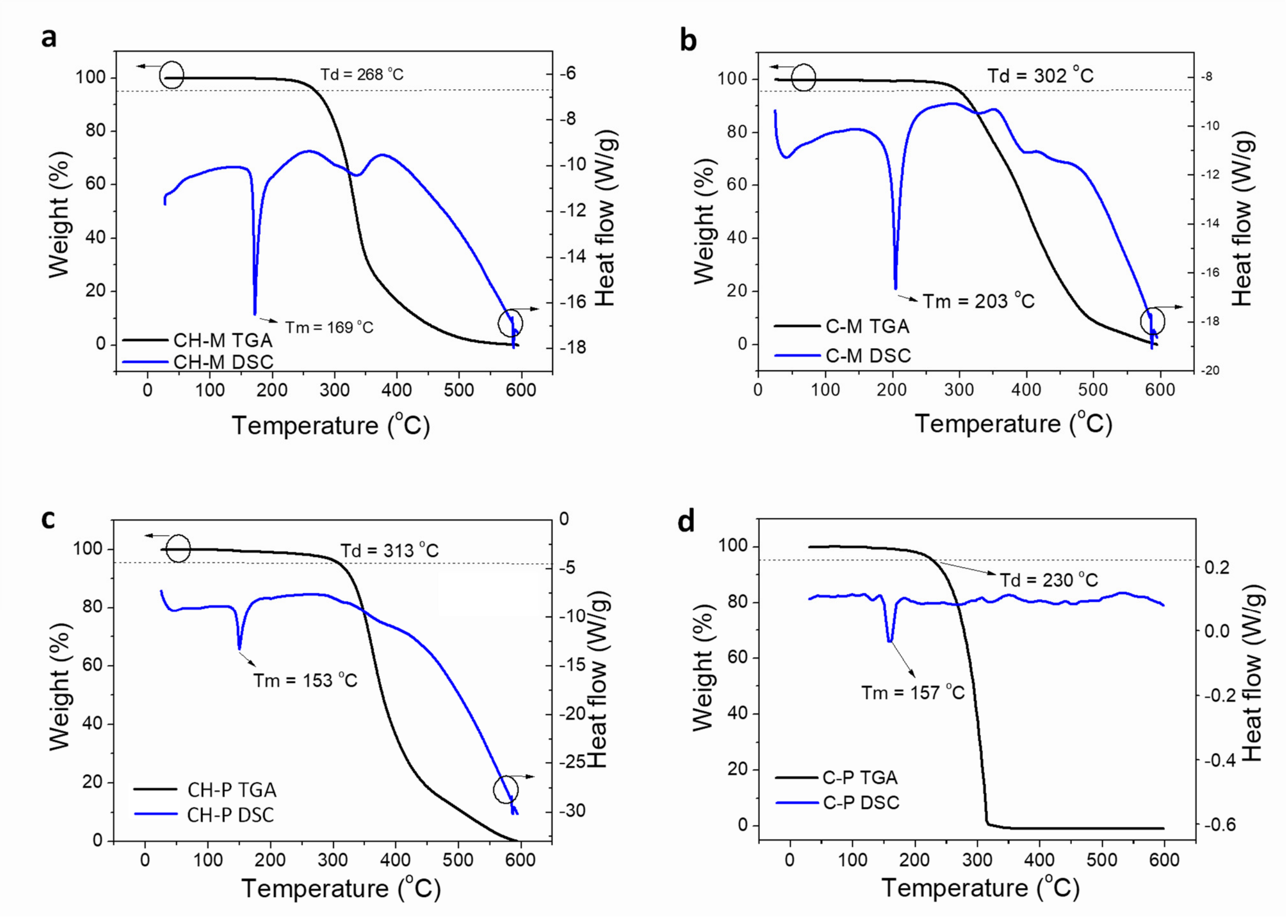

Supplementary Fig. 22 Thermogravimetric analysis (TGA) and differential scanning calorimetry (DSC) results of **a** CH-M, **b** C-M, **c** CH-P, and **d** C-P.

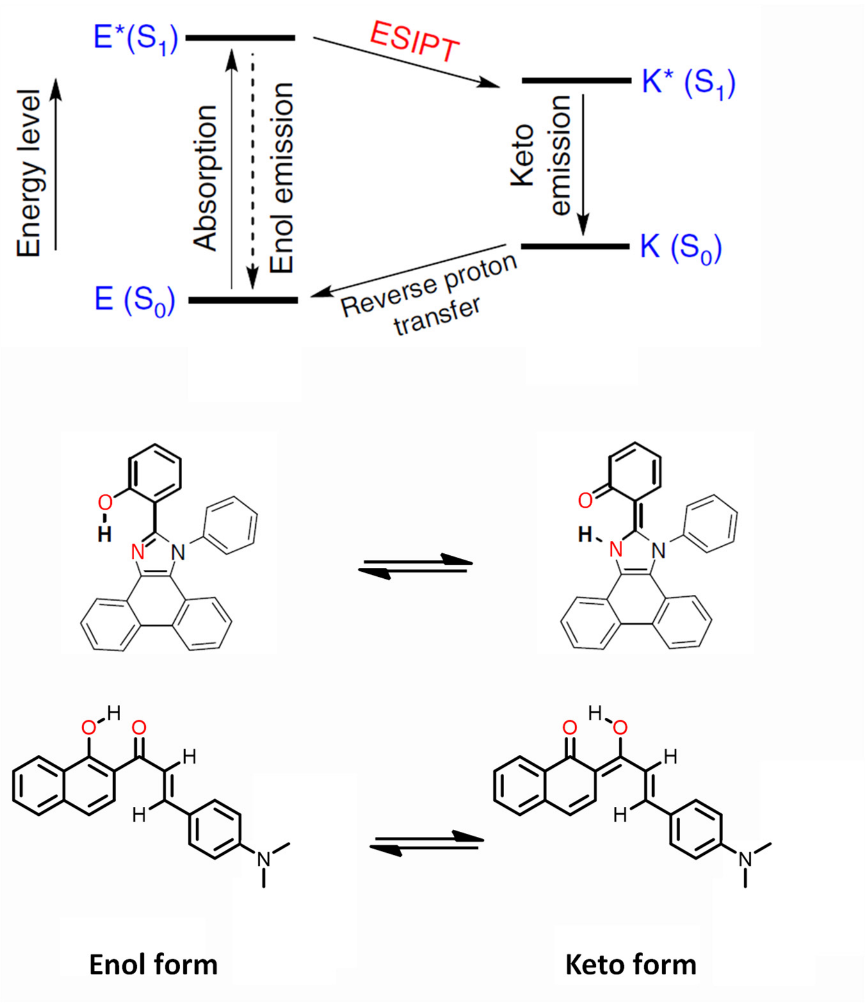

Supplementary Fig. 23 Schematic representation of four-level photocycle of the ESIPT process.

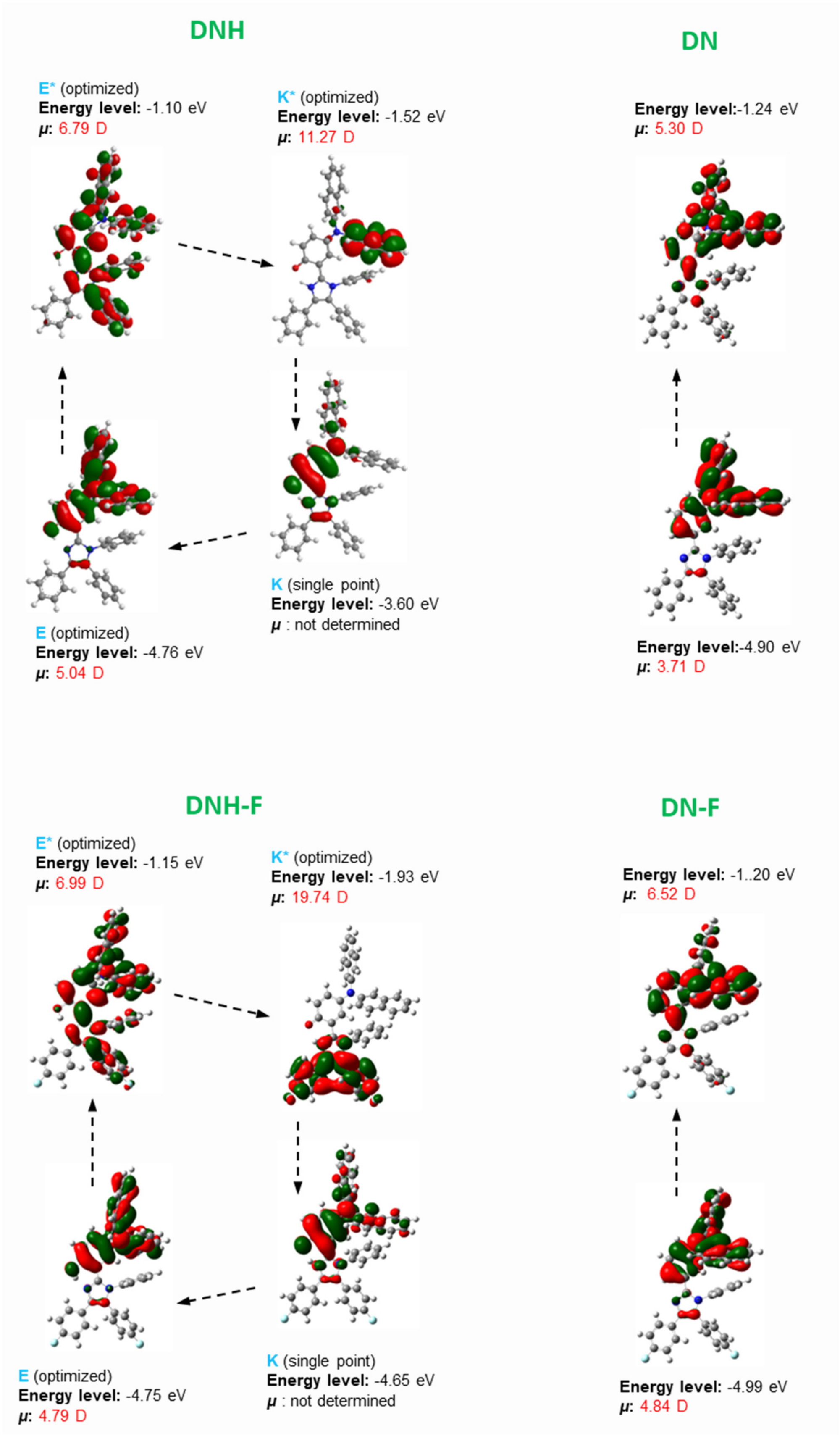

Supplementary Fig. 24 The orbital diagrams of UV-responding ESIPT-molecules (DNH and DNH-F in enol and keto forms) and non-ESIPT-molecule (DN and DN-F), calculated by DFT. Theoretically calculated dipole moment values and energy levels are added.

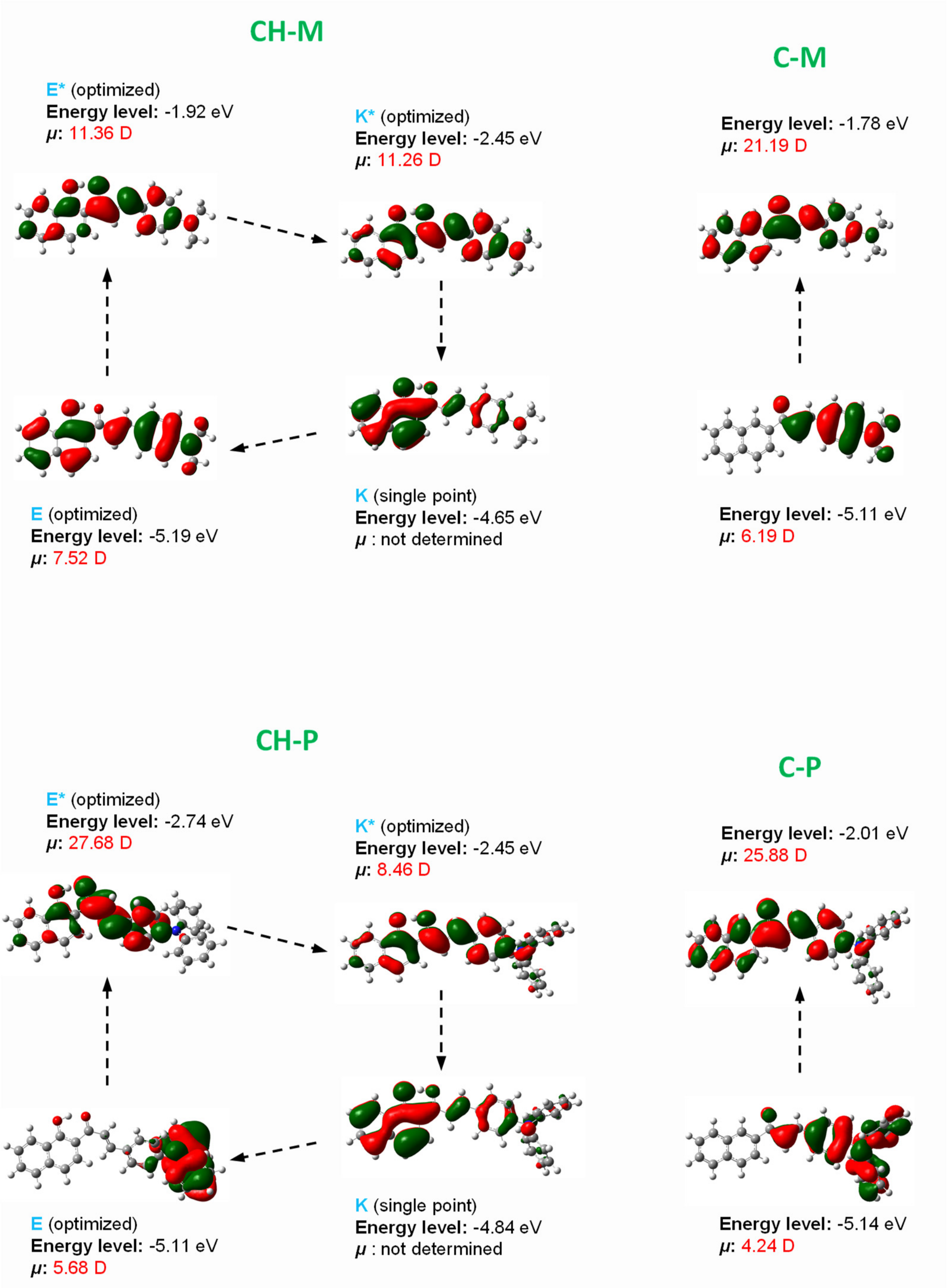

Supplementary Fig. 25 The orbital diagrams of visible-light-responding ESIPT-molecule (CH-M and CH-P in enol and keto forms) and non-ESIPT-molecule (C-M and C-P), calculated by DFT. Theoretically calculated dipole moment values and energy levels are added.

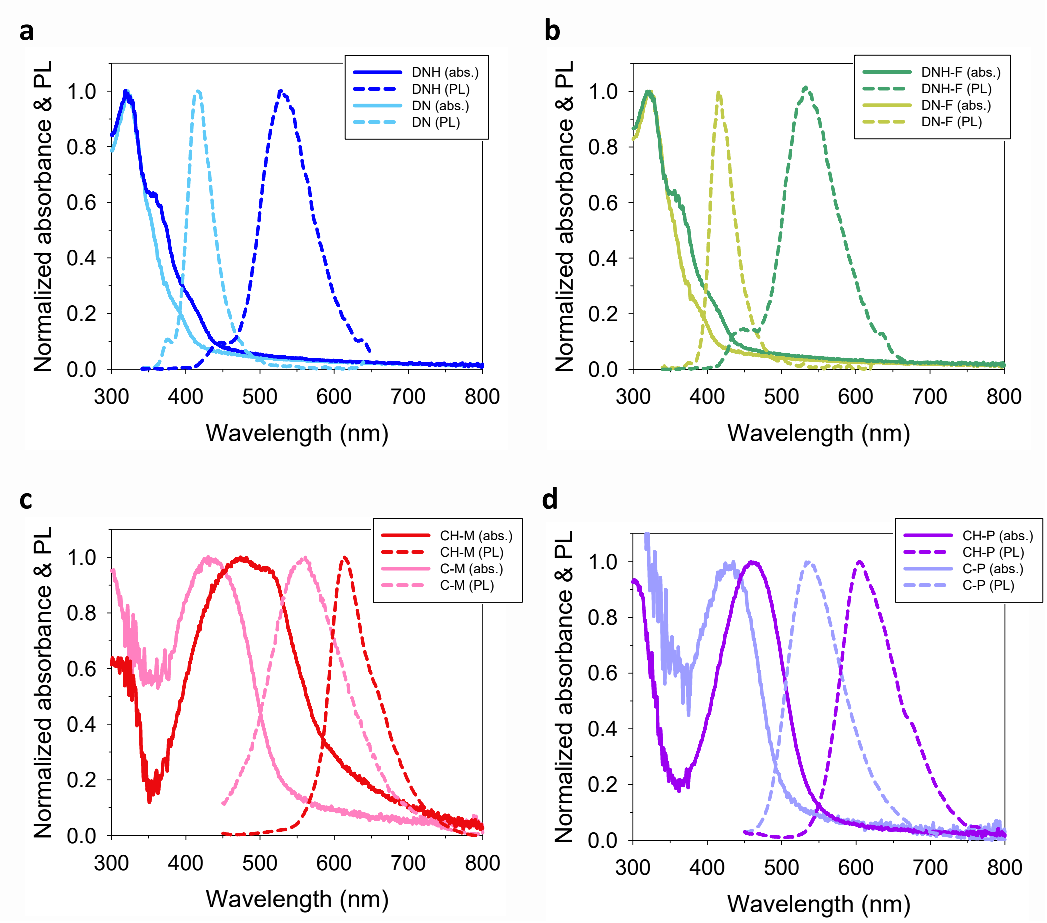

Supplementary Fig. 26 Absorbance and steady-state photoluminescence of the organic semiconductor thin films casted on quartz: (a) DNH and DN, (b) DNH-F and DN-F, (c) CH-M and C-M, and (d) CH-P and C-P.

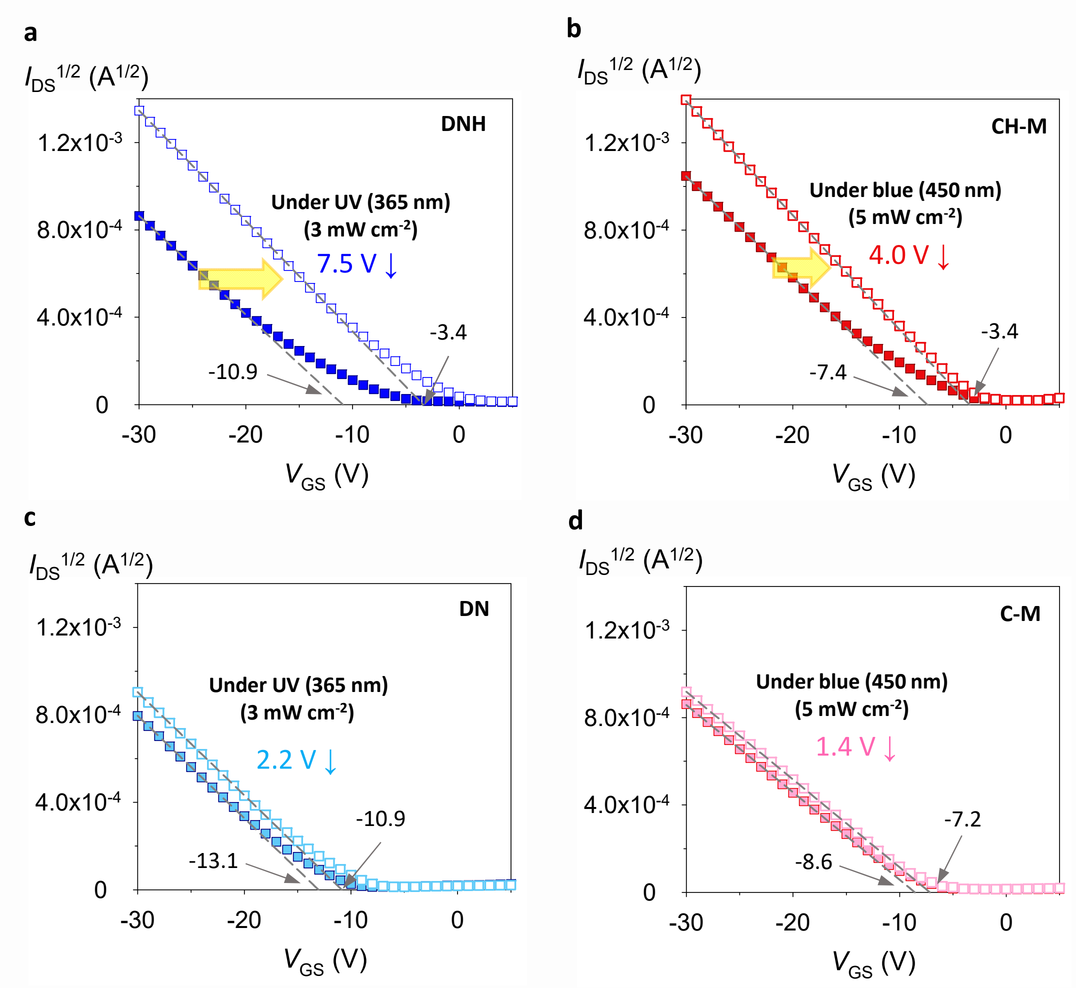

Supplementary Fig. 27 Transfer characteristics of OFETs (I_{DS} vs. V_{GS}) at V_{DS} = -15 V at dark and light-irradiation condition: **a**,**b** with ESIPT-active organic thin film as gate dielectric (a: DNH and b: CH-M). **c**,**d** with non-ESIPT organic thin film as gate dielectric (c: DN and d: C-M).

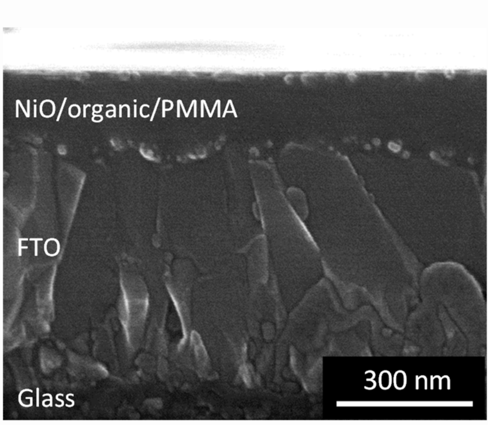

Supplementary Fig. 28 Cross-sectional SEM image of memristor without top electrode (glass/FTO/NiO/organic/PMMA).

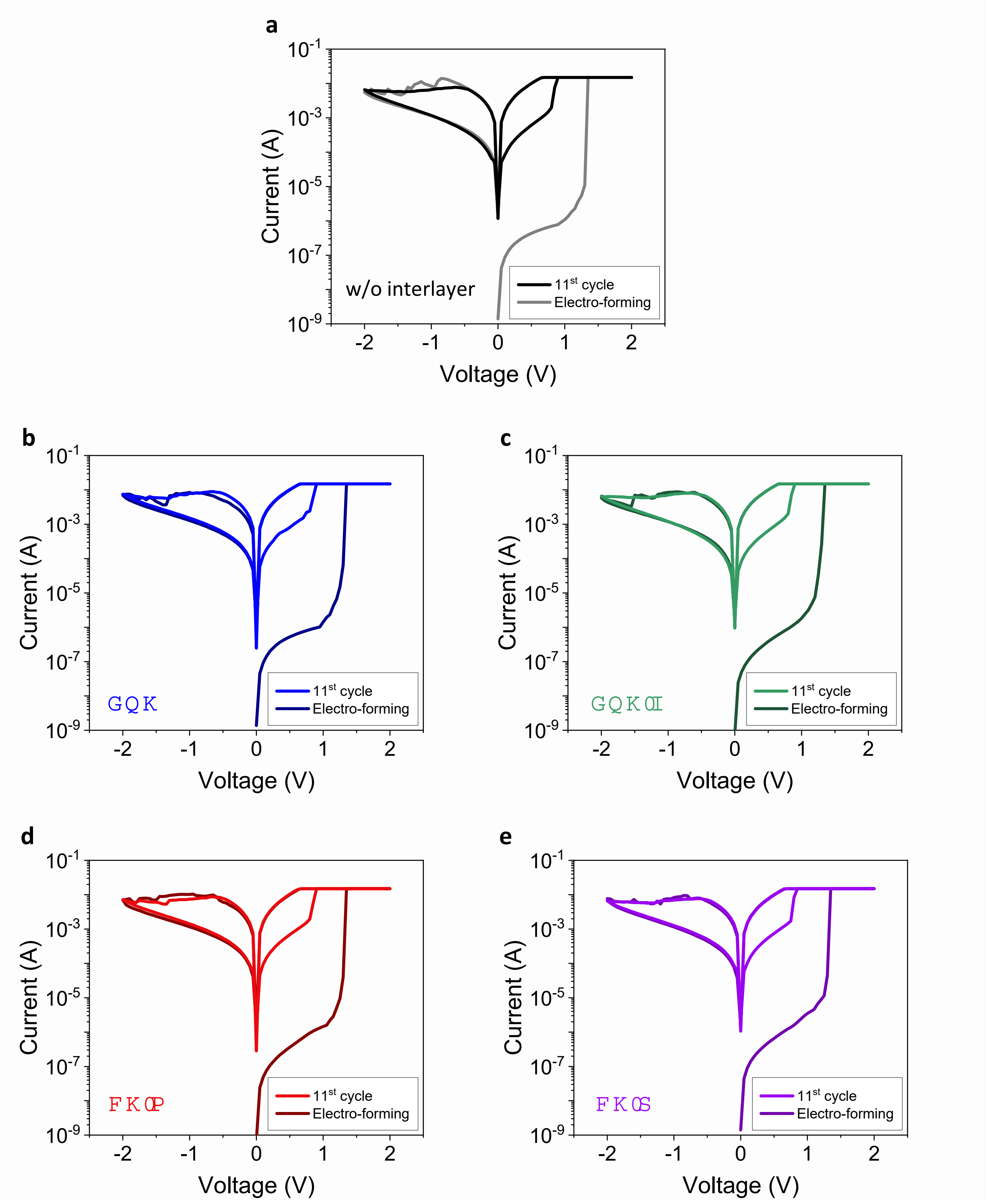

Supplementary Fig. 29 Measured 1st and 11th cycle d.c. *I–V* characteristics of the memristor with different organic semiconductor (glass/FTO/NiO/PMMA/orgnic layer/Ag). Positive voltage bias is applied to top Ag electrode (FTO: grounded). **a** Without organic layer. **b-e** With organic layer (b: DNH, c: DNH-F, d: CH-M, and e: CH-P).

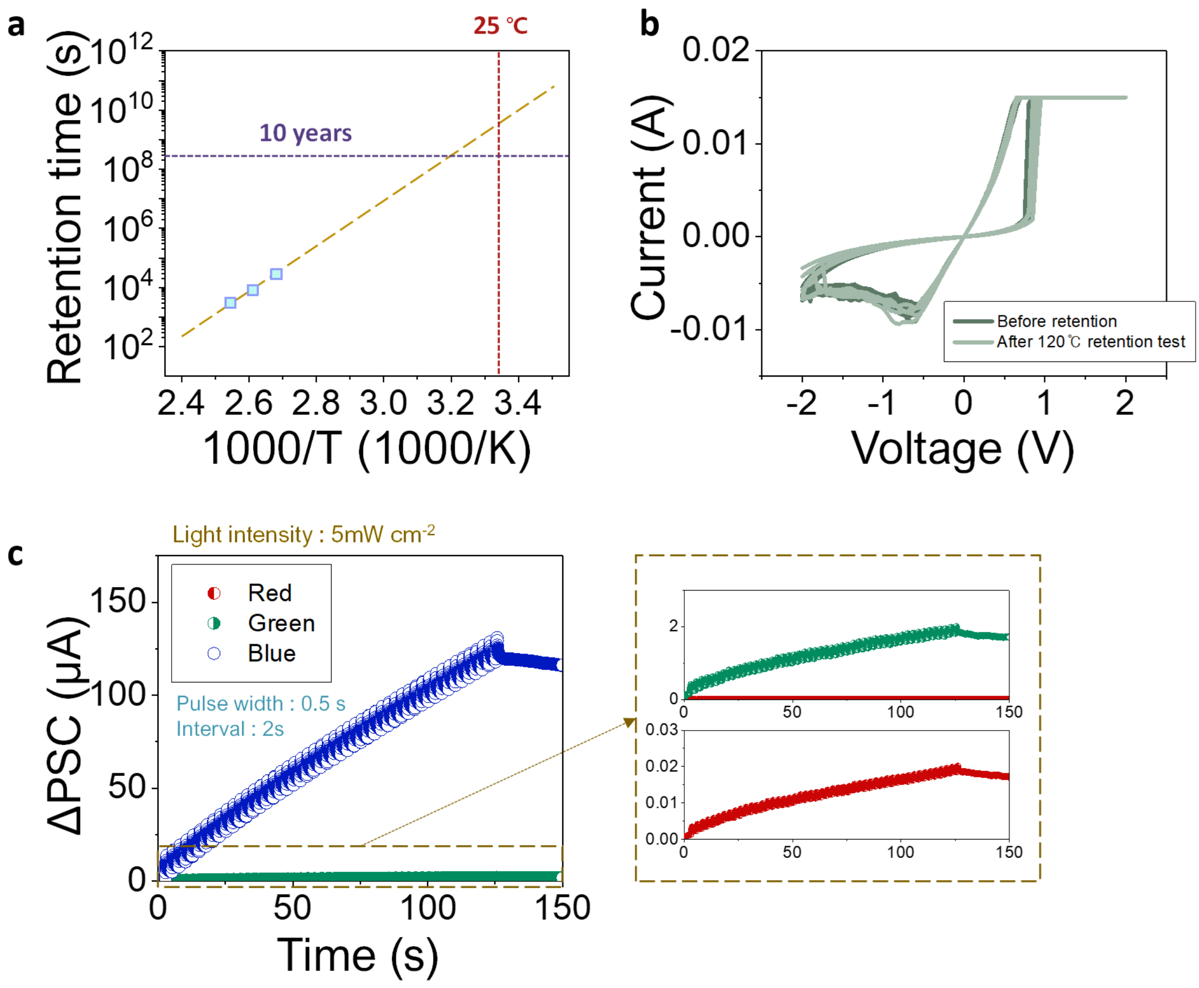

Supplementary Fig. 30 **a** Plots for retention of high temperature fitted to Arrhenius plot. **b** D.C *I*-*V* sweep cycles before and after 120 °C retention test. **c** Light responsivity after 120 °C retention test.

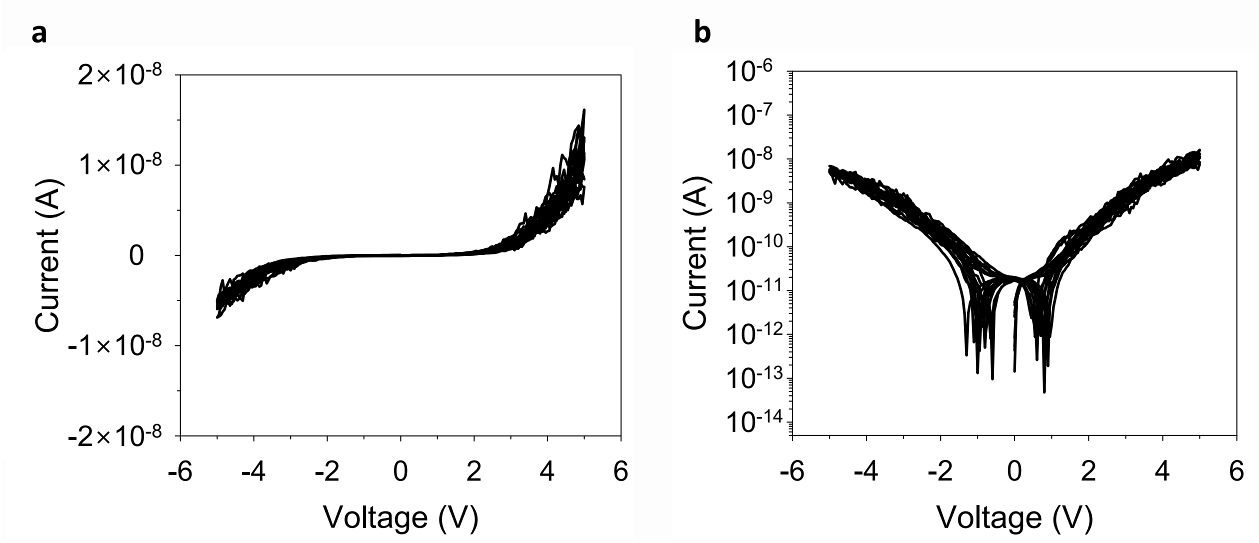

Supplementary Fig. 31 Measured 10-cycle d.c. *I–V* characteristics of the memristor built with Au top electrode (glass/FTO/NiO/PMMA/Au). Positive voltage bias is first applied to top Au electrode (FTO: grounded). **a** Normal scale. **b** Log scale.

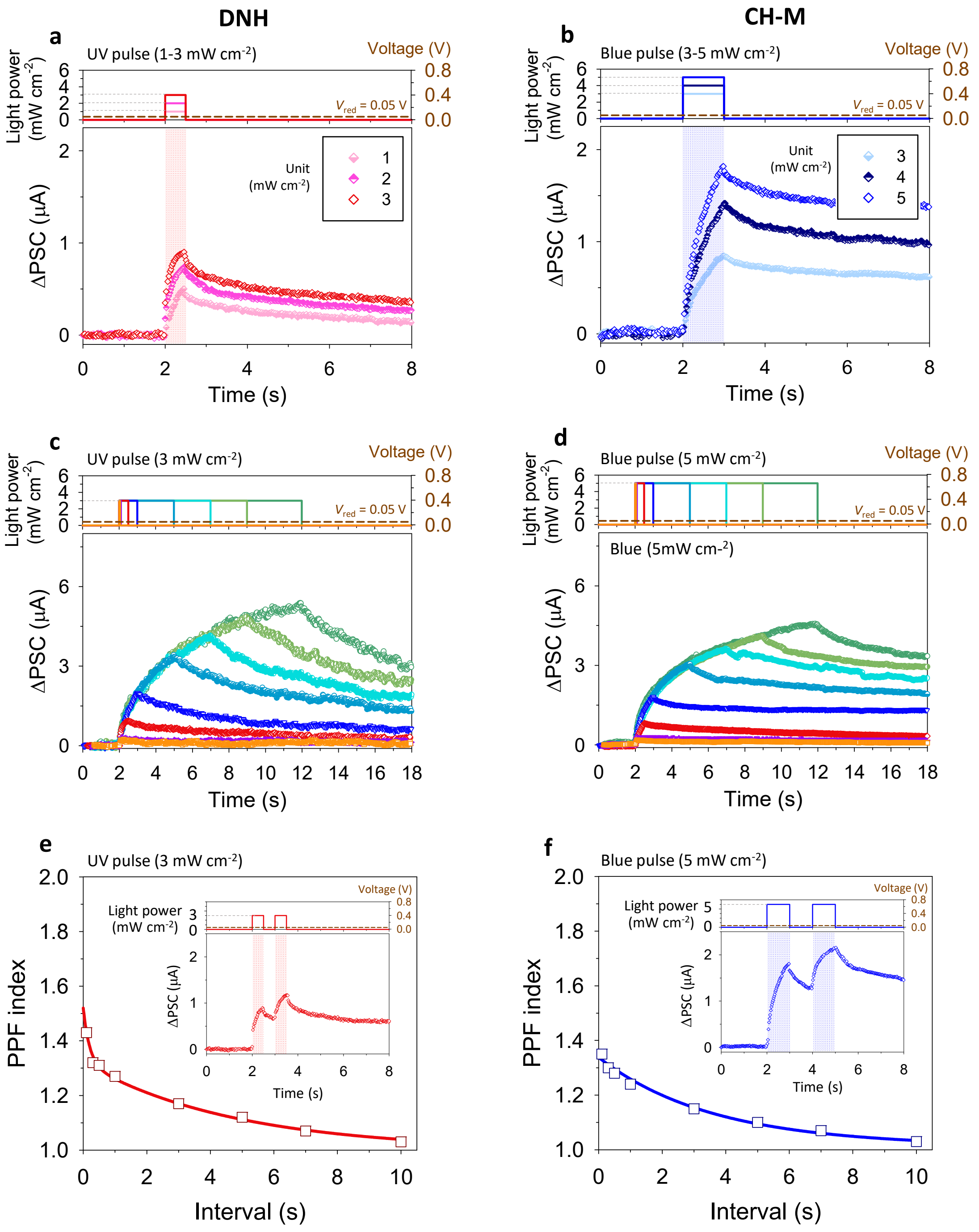

Supplementary Fig. 32 a-d EPSC responses of the memristors integrated with various organic thin films: (a,c) DNH and (b,d) CH-M. Light pulse conditions are illustrated in top figures. Read voltage is 0.05 V. (a,b) Depending on the amplitude of the light pulse (pulse widths are fixed): UV pulse (365 nm and 0.5 s width) and blue light pulse (450 nm and 1 s width) are used for the memristor with DNH and that with CH-M. (c,d) Depending on the width of the light pulse (pulse amplitudes are fixed): UV pulse (365 nm and 3 mWcm^{-2}) and blue light pulse (450 nm and 5 mWcm^{-2}) are used for the memristor with DNH and that with CH-M. **e,f** PPF index as a function of interval between two consecutive pulses (Δt). Read voltage is 0.05 V. (e) Amplitude and width of UV pulse (365 nm) for the memristor with DNH are 3 mW cm^{-2} and 0.5 s. (f) Those of blue light pulse (450 nm) for the memristor with CH-M are 5 mW cm^{-2} and 1 s. Bottom inset figures are about PPF behaviors by a pair of presynaptic pulses for 0.5 s (DNH) and 1 s (CH-M) interval cases. Top inset figures illustrate the applied light pulse conditions.

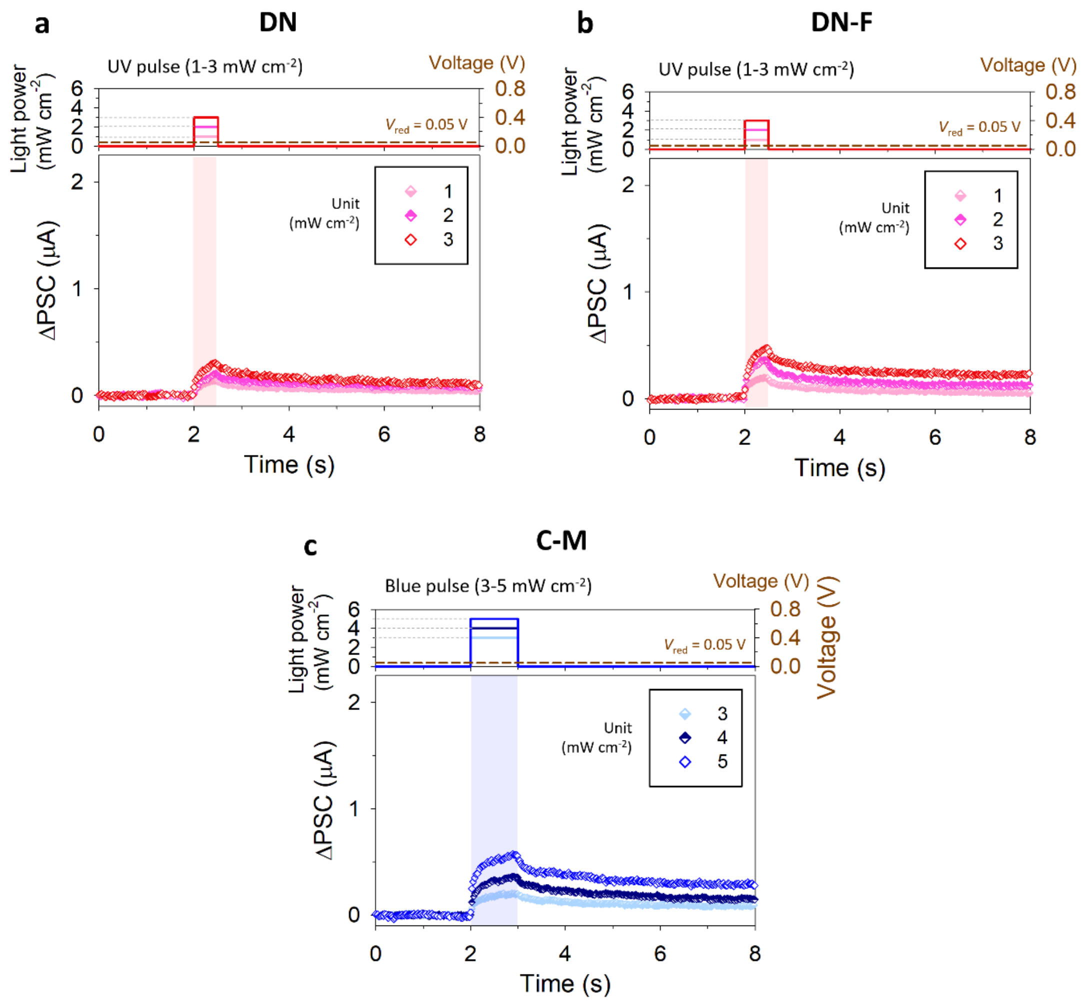

Supplementary Fig. 33 EPSC responses of the memristors with various organic thin films depending on the amplitude of the light pulse (pulse widths are fixed): UV pulse (365 nm and 0.5 s width) and blue pulse (450 nm and 1 s width) are used for the memristor with DN (or DN-F) and that with C-M. Read voltage is 0.05 V. **a** DN, **b** DN-F, and **c** C-M.

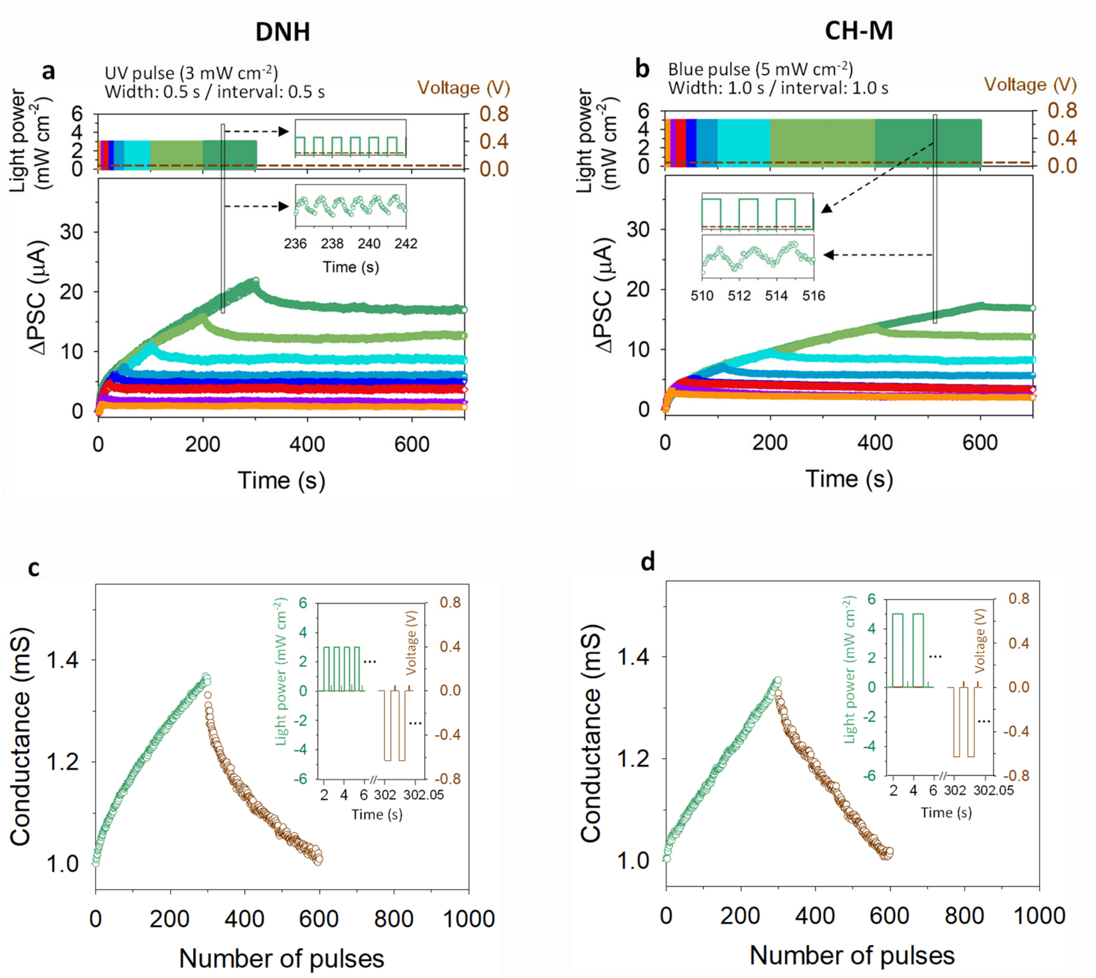

Supplementary Fig. 34 a,**b** Pulse number-dependent PSC: (a) DNH and (b) CH-M. In inset figures, x-axis about time is enlarged. Light pulse conditions are depicted in top figures. UV pulse (365 nm, power: 3 mWcm^{-2}, width: 0.5 s, and interval: 0.5 s) and blue light pulse (450 nm, power: 5 mWcm^{-2}, width: 1 s, and interval: 1 s) are used for the memristor with DNH and that with CH-M. Read voltage is 0.05 V. **c**,**d** LTP/LTD characteristics: (c) DNH and (d) CH-M. Light and voltage pulse conditions for LTP and LTD are depicted in inset figures. Pulse conditions of potentiation(P)/depression(D) of the memristor with DNH are 365 nm, 3 mWcm^{-2} power, 0.5 s width, and 0.5 s interval/-0.63 V, 0.01 s width, and 0.013 s interval. Those of the memristor with CH-M are 450 nm, 5 mWcm^{-2} power, 1 s width, and 1 s interval/-0.63 V, 0.01 s width, and 0.013 s interval. Amplitude and width of the read voltage pulse are 0.05 V and 0.001 s.

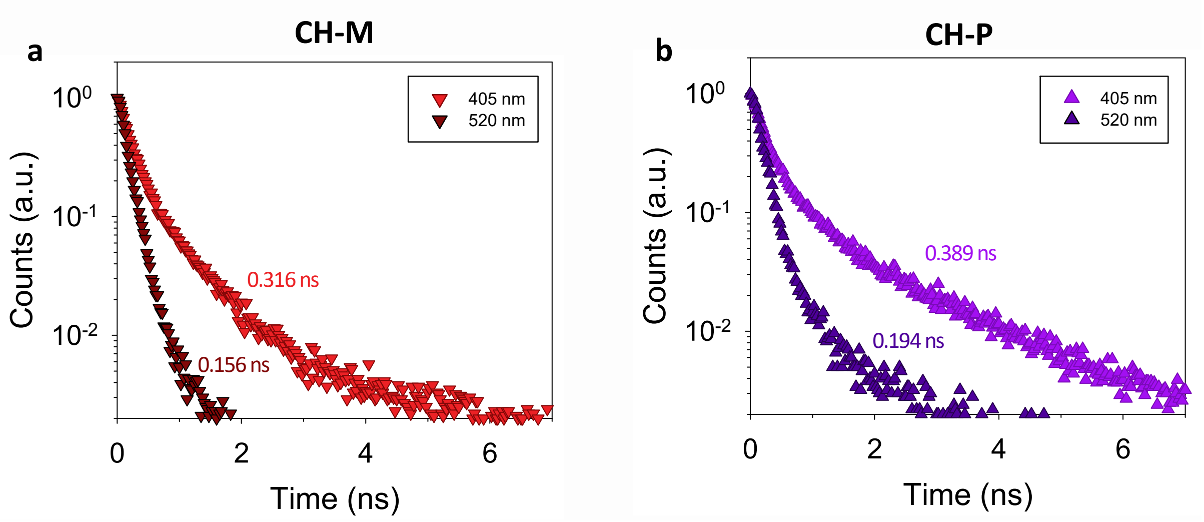

Supplementary Fig. 35 Time-resolved photoluminescence of organic semiconductor thin films casted on quartz excited at 405 and 520 nm wavelengths. **a** CH-M (τ_{avg}: 3.16 $\times 10^{-10}$ s at 405 nm, 1.56 $\times 10^{-10}$ s at 520 nm). **b** CH-P (τ_{avg}: 3.89 $\times 10^{-10}$ s at 405 nm, 1.94 $\times 10^{-10}$ s at 520 nm).

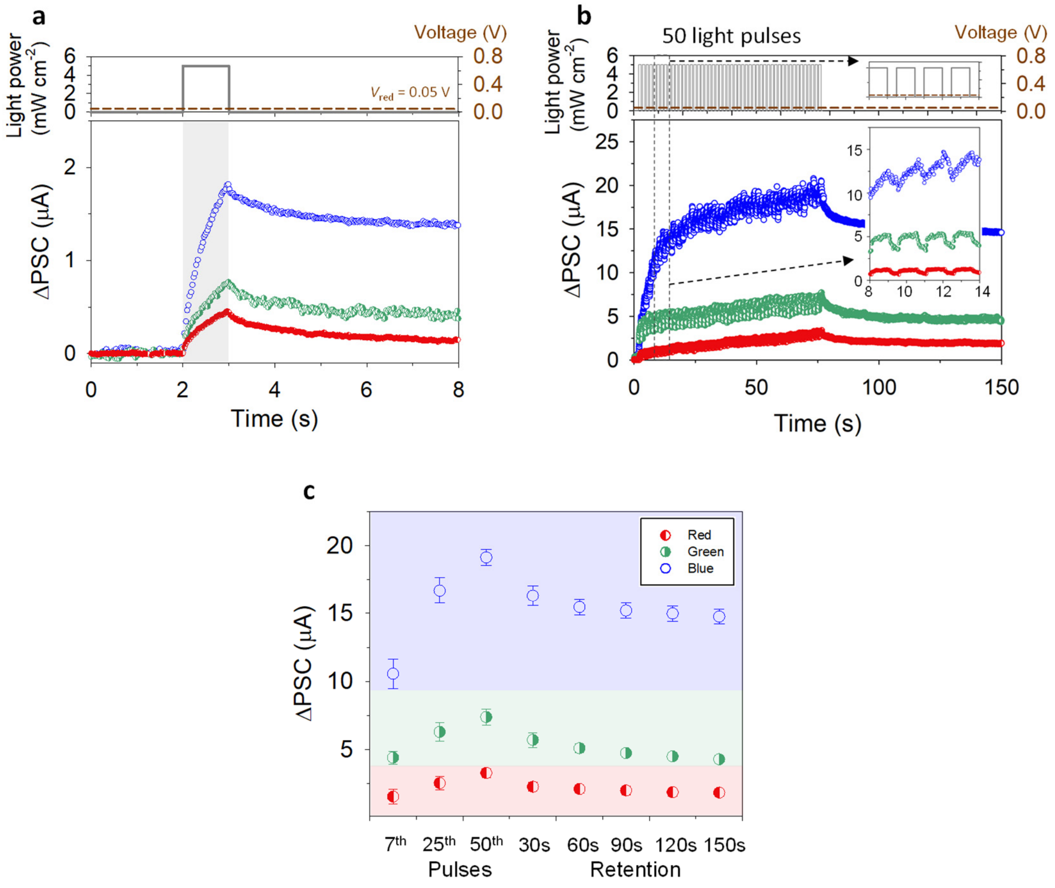

Supplementary Fig. 36 a EPSC responses of CH-M-integrated memristor under R-, G-, and B-light pulse irradiation (450, 525, and 630 nm) having the same pulse condition (5 mWcm^{-2} power and 1 s width). Light pulse condition is depicted in top figure. Read voltage is 0.05 V. **b** PSC variations of CH-M-integrated memristor under 50 consecutive pulses of R-, G-, and B-light (450, 525, and 630 nm) having the same pulse conditions (5 mWcm^{-2} power, 1 s width, and 0.5 s interval). Light pulse condition is depicted in top figure. Read voltage is 0.05 V. In inset figures, x-axis about time is enlarged. **c** PSC distribution depending on the number of pulses and retention time, obtained from 8×8 pixels, for color discrimination. Circles and error bars represent average and standard deviation.

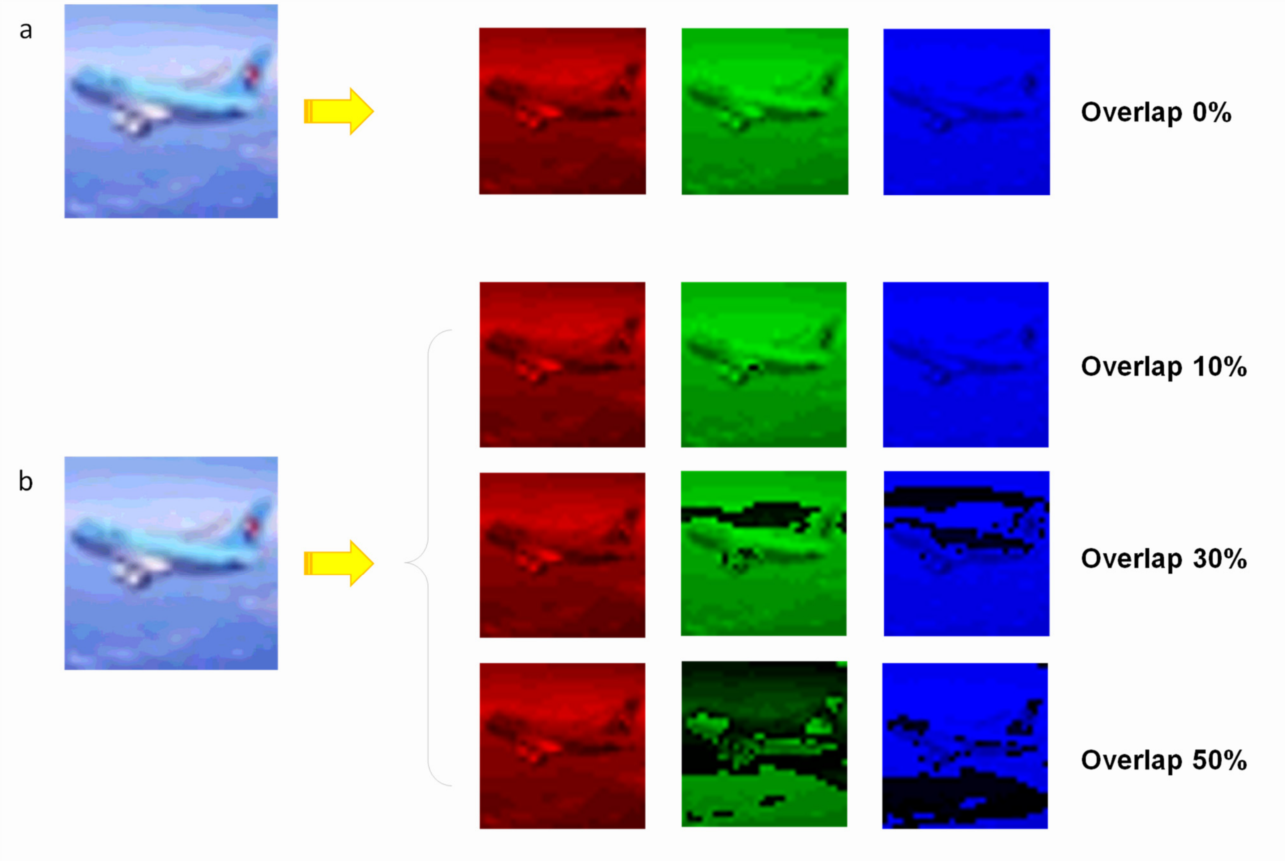

Supplementary Fig. 37 Pre-processing results of an exemplary CIFAR-10 dataset image [4-bit resolution: (R, G, B) = (0~15, 0~15, 0~15)]. **a** Color input image is separated into RGB three channels without overlapping. **b** Exemplary RGB channels, where the overlapped RGB components reach 10%, 30%, and 50 %.

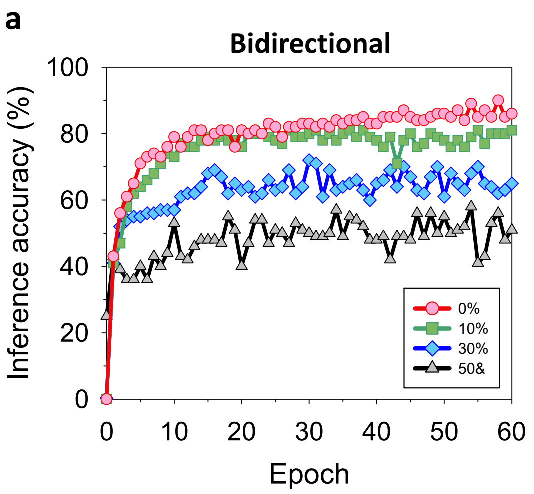

Supplementary Fig. 38 Image recognition rate of the CIFAR-10 dataset after pre-processing using the optoelectronic memristors with a full 8-bit resolution RGB color-separating capability. The percentage values represent the overlapped RGB components. **a** Bidirectional weight update. **b** Unidirectional weight update.

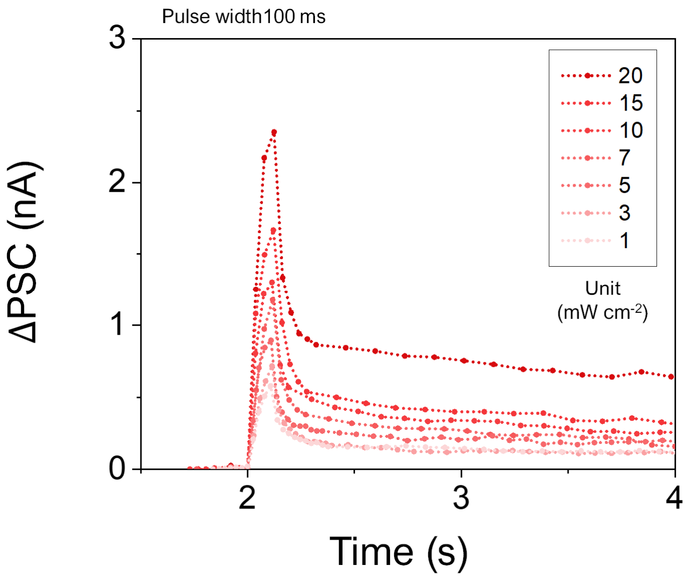

Supplementary Fig. 39 Short-term plasticity characteristic of CH-P-integrated memristor depending on light intensity (1 – 20 mW cm^{-2}) with the pulse width of 100 ms.

Supplementary Table 1 The device structure and performances of optoelectronic synapses in literature. Nonlinearity and symmetricity of the devices in literature are estimated by extracting data from LTD/LTP plots in each reference using the equations in Supplementary Note 10.

	Device structure	Light wavelength	Nonlinearity		Dynamic Range (on/off)	Symmetricity	Pulse number (LTP/LTD)	CCV cycle	Ref. in SI
			LTP	LTD					
	**FTO/oxide/CH-P/PMMA/Ag(Cu)**	**Visible: 450<br>Visible: 525<br>Visible: 630**	**0.5**	**0.7**	**5.6**	**54.0**	**300 / 300**	**110,000**	**This work**
2T	Si/SiO_2/Pd/MoO_x/ITO	UV : 365	1.9	-	2.1	-	500 / -	-	1
	ITO/SnO_2/$CsPbCl_3$/TAPC/TAPC:MoO_3/MoO_3/Ag	UV : 365	1.7	-	1.7	-	10 / -	-	2
	Glass/ITO/PEDOT:PSS/CuSCN/$CsPbBr_3$/Au	UV : 365	1.7	0.5	2.8	10.3	20 / 20	-	3
	Au/$La_{1.875}Sr_{0.125}NiO_4$/Au	UV : 365	2.0	-	8.5	-	50 / -	1000	4
	Glass/ITO/MoS_2/TiN_xO_{2-x}/Al	UV : 365	4.2	-	8.0	-	300 / -	-	5
	Si/SiO_2/$CsPbBr_3$/Al_2O_3/Pentacence/Au	UV : 365	2.1	-	5.3	-	100 / -	-	6
	PDMS/Al/Mxene-ZnO/ITO	UV : 365	1.0	1.1	3.9	22.5	50 / 50	400	7
	Glass/ITO/Bi_2FeMnO_6/Au	UV : 365	0.3	0.3	4.1	51.0	50 / 50	-	8
	SLG/WS_2/SLG	UV : 400	0.1	-	14.4	-	100 / -	-	9
	Glass/Ag-TiO_2/Au	UV : 360<br>Visible: 532	1.7	4.0	12.0	4.0	50 / 50	-	10
	Glass/ITO/PFTPA-AZO/Au	UV : 365<br>Visible: -	0.3	0.3	76.5	133.0	32 / 32	-	11
	Glass/ITO/$Cs_2AgSbBr_6$/Au	Visible: 400	1.4	-	3.6	-	30 / -	-	12
	Glass/ITO/ZTO/ITO	Visible: 405	4.0	1.4	1.5	-	200 / 100	12,000	13
	Glass/ITO/P3HT/Au	Visible: 445	2.1	-	1.2	-	100 / -	-	14
	Glass/ITO/PEDOS:PSS/CuSCN/TFB/CdSe/QDs/ZnO / Ag	Visible: 452	5.0	2.6	3.0	2.5	200 / 200	-	15
	Glass/$CuAlO_2$@ZnO nanorods/ITO	Visible: 450	1.3	2.0	11.9	19.0	32 / 32	-	16
	Glass/ZnO/P3HT/Au	Visible: 520	2.3	-	5.3	-	100 / -	-	17
	ITO/In-doped TiO2/Au	Visible: 530	1.4	1.0	12	29.0	12 / 12	-	18
	Au/$MAPbBr_3$/Au	Visible: 532	3.3	3.3	7.5	1.02	5 / 5	320	19
	Si/ReS_2/h-BN/Au	Visible: 532	-0.2	-	11.4	-	32 / -	-	20
	Quartz plate/MoS_2/In/Au	Visible: 550	1.7	-	4.1	-	300 / -	-	21
	Si/SiO_2/In_2S_3/Au	Visible: 635	1.4	-	1.35	-	43 / -	200	22
	Glass/ITO/Cu_3P nanoribbons/Ag	Visible: 660	0.5	3.9	15.8	4.0	20 / 20	100	23
	Glass/CuPc/CuI	Visible: 660	5.3	-	4.8	-	100 / -	-	24
	Pt/oxygen-deficient IGZO (O_D-IGZO)/oxygen-rich IGZO (O_R-IGZO)/Au	Visible: 420<br>IR : 800	5.0	1.7	1.6	4.4	100 / 100	-	25
	Glass/ITO/TiS_3/Al	Visible: 400<br>Visible: 530<br>IR : 808	1.4	1.3	1.7	8.3	11 / 11	-	26
	Si/Pyr-GDY/Gr/PbS-QD/Au	Visible: 450<br>IR : 980	1.7	1.1	3.1	8.4	150 / 150	-	27
	ITO/Nb:$SrTiO_3$/Silver paste	Visible: 528<br>Visible: 630	2.9	5.0	2.3	3.0	100 / 100	-	28
	Si/SiO_2/Au/MoO_3/Au	IR : 785	7.1	-	10.0	-	40 / -	-	29
	Si/SiO_2/PBTT/Au	IR : 1342	-	-	-	-	-	-	30
	ITO/ZnO/Ag	White light	11.1	1.4	25.0	2.8	25 / 25	500	31
	ITO/PbS QDs/TiN	Visible : unknown	4.3	-	10^5	-	10	-	32
3T	Si/SiO_2/NT-CN/PMMA/ Pentacene/Au	UV : 365	0.8	1.1	20.0	3.9	30 / 30	-	33
	Si/SiO_2/Silk&CDs/Pentacene/Au	UV : 365	5.0	-	15.6	-	100 / 100	500	34
	Si/SiO_2/C8-BTBT/C8-BTBT/Au	UV : 365	1.0	-	4.0	-	10 / -	-	35
	Si/SiO_2/In-Ga-Zn-O/Al	UV : 365	-1.4	-	18.0	-	50 / -	-	36
	Si/SiO_2/p-6P/CuPc/Au	UV : 365	0.8	-1.3	48.2	4.3	85 / 85	-	37
	Si/SiO_2/C8-BTBT/PS/QDs/Au	UV : 365	1.7	1.3	38.0	3.8	100 / 100	50	38
	WBG III-IV Materials	UV : 365	3.3	2.5	37.0	2.8	100 / 100	-	39
	Si/SiO_2/PO_x/BP/PO_x/Au	UV : 280<br>UV : 365<br>Visible: 660	1.7	-	1.3	-	15 / -	-	40
	Si/SiO_2/ZB NPs/pentacene/Au	UV : 365<br>Visible: 520<br>Visible: 660	0.2	1.7	1.4	8.1	25 / 25	-	41
	Si/SiO_2/SWCNT + PF-b-PI/Au	Visible: 405	1.1	1.3	1.7	2.4	45 / 45	120	42
	Si/SiO_2/HfO_2/P3HT/Au	Visible: 405	2.0	-	3.0	-	10 / -	-	43
	Si/SiO_2/Graphene/PQD/Ni	Visible: 430	2.9	2.5	28,800.0	2.3	20 / 20	-	44
	Si/SiO_2/PVP/$CsBi_3I_{10}$/PDPP4T/Au	Visible: 430	1.4	2.5	37.6	3.4	50 / 50	-	45
	Si/SiO_2/Grahene/DTAP/Au	Visible: 430	3.7	-	1.0	-	20 / -	-	46
	Si/SiO_2/Si_3N_4/Porphyrin/Ti/Al	Visible: 435	1.7	-	1.2	-	100 / -	50	47
	Si/SiO_2/IGZO/$CsPbBr_3$/ITO/PMMA	Visible: 445	1.0	-	1.7	-	200 / -	-	48
	Si/SiO_2/MoS_2/ Ni	Visible: 450	2.5	3.3	8.0	3.7	50 / 50	-	49
	Au/ICCN/$CsPbBr_3$ QDs + DPP-DTT/Au	Visible: 450	0.7	1.7	6.2	6.6	20 / 20	150	50
	Si/SiO_2/h-BN/MoS_2&Graphene flake/Cr/Au	Visible: 520	-2.0	1.7	45.0	-	18 / 30	-	51
	Glass/Cr/Al_2O_3/a-IGZO/CdS/ITO	Visible: 520	0.8	-	-	-	100 / -	-	52

	Si/SiO_2/Si NM/$MAPbI_3$/PMMA/Au	Visible: 532	3.4	-	2.5	-	100 / -	-	53
	Si/SiO_2/SWCNTs/Chlorophyll-a/Au/Cr	Visible: 665	2.5	-	7.0	-	100 / -	-	54
	$Si/SiO_2/In_2Se_3/WSe_2$/Pd	Visible: 750	5.0	-	2.0	-	200 / -	-	55
	Si/SiO_2/P3HT&QD hybrid solution/Au	Visible: 405<br>Visible: 450	0.8	-	6.0	-	50 / -	-	56
	Si/SiO_2/SWCNT(S)/$CsPbBr_3$/PDPP4T/PEDOT:PSS (D)	Visible: 450<br>Visible: 550<br>Visible: 650	1.4	-	2.1	-	20 / -	-	57
	$Si/SiO_2/(PEA)_2SnI_4$/Au	Visible: 465<br>Visible: 525<br>Visible: 625	10.0	-	3.7	-	200 /	-	58
	$Si/Au/Al_2O_3/WSe_2/Al_2O_3/WSe_2$/Au	Visible: 473<br>Visible: 532<br>Visible: 655	0.5	2.5	3.2	0.9	20 / 20	-	59
	Si/SiO_2/TIN/$HfZrO_x$/IGZO/Al	White light	1.0	-	2.8	-	10 / -	-	60

Supplementary Table 2 Fitted parameters of PL decay curves of organic molecule thin films on quartz samples (405 nm wavelength excitation).

	A_1 (%)	τ_1 (10^{-10} s)	A_2 (%)	τ_2 (10^{-10} s)	τ_{avg} (10^{-10} s)
DNH	78	2.68	22	12.83	4.93
DN	91	1.75	9	9.22	2.42
DNH-F	73	3.47	27	16.93	7.10
DN-F	91	1.77	9	9.55	2.47
CH-M	85	2.02	15	9.80	3.16
C-M	99	1.54	1	9.10	1.55
CH-P	86	2.32	14	13.50	3.89
C-P	99	1.55	1	9.21	1.60

where, $\text{Counts}(t) = A_1\exp(-t/\tau_1) + A_2\exp(-t/\tau_2)$

τ_{avg}: amplitude weighted average lifetime

Supplementary Table 3 Fitted parameters of PPF index.

	C_1	τ_1 (s)	C_2	τ_2 (s)
DNH	0.2133	0.39	0.1256	44.04
DNH-F	0.4036	0.53	0.1292	46.60
CH-M	0.1734	1.03	0.1186	69.58
CH-P	0.2349	0.27	0.8402	45.37

where, $\text{PPF index}(\Delta t) = C_1 \exp(-\Delta t/\tau_1) + C_2 \exp(-\Delta t/\tau_2) + 1$

Supplementary Table 4 Average and standard deviation of each pulse stream condition obtained from the measurement results of 10 devices.

Pulse stream pattern	Average (nA)	Standard deviation (pA)
0000	0.00	0.00
0001	0.20	0.64
0010	0.07	0.69
0011	0.25	0.61
0100	0.04	0.58
0101	0.24	3.60
0110	0.11	1.60
0111	0.29	0.81
1000	0.02	0.33
1001	0.23	2.10
1010	0.09	0.48
1011	0.30	0.80
1100	0.08	0.32
1101	0.27	0.81
1110	0.16	0.99
1111	0.32	1.60

Supplementary Note 1 Detailed ^{1}H NMR, ^{13}C NMR and MS data of 4-(di(naphthalen-2-yl)amino)-2-(1,4,5-triphenyl-1H-imidazol-2-yl)phenol (**DNH**).

^{1}H NMR (400 MHz, $CDCl_3$, 25 °C, TMS) δ (ppm): 13.68 (s, 1H), 7.75 (d, J = 7.9 Hz, 2H), 7.62 (d, J = 8.8 Hz, 2H), 7.57 – 7.51 (m, 4H), 7.38 (dt, J = 19.4, 7.2 Hz, 4H), 7.26 (dt, J = 13.6, 7.0 Hz, 3H), 7.13 (ddd, J = 1.8, 10.4, 4.6 Hz, 9H), 7.04 (d, J = 6.8 Hz, 2H), 6.85 (d, J = 7.8 Hz, 2H), 6.46 (t, J = 7.7 Hz, 2H), 6.35 (t, J = 6.3 Hz, 2H). **^{13}C NMR** (100 MHz, $CDCl_3$, 25 °C, TMS) δ (ppm): 155.56, 145.55, 144.42, 138.22, 136.17, 134.98, 134.46, 132.97, 131.16, 130.64, 129.72, 129.54, 129.13, 128.60, 128.36, 128.32, 127.74, 127.47, 127.07, 126.89, 126.80, 126.22, 124.35, 124.19, 123.72, 118.73, 113.77. **LC-MS** (ESI): [$C_{47}H_{33}N_3O$] $[M]^+$ 656.26, found; 656.40.

Supplementary Note 2 Detailed ^{1}H NMR, ^{13}C NMR and MS data of N-(naphthalen-2-yl)-N-(3-(1,4,5-triphenyl-1H-imidazol-2-yl)phenyl)naphthalen-2-amine (**DN**).

^{1}H NMR (400 MHz, $CDCl_3$, 25 °C, TMS) δ (ppm): 7.76 (d, J = 7.7 Hz, 2H), 7.67 (d, J = 8.8 Hz, 2H), 7.57 (t, J = 7.7 Hz, 4H), 7.51 (d, J = 7.8 Hz, 1H), 7.44 – 7.34 (m, 4H), 7.28 (s, 3H), 7.22 (d, J = 7.6 Hz, 2H), 7.20 – 7.11 (m, 7H), 7.06 (d, J = 6.4 Hz, 2H), 6.97 (s, 1H), 6.90 (d, J = 6.1 Hz, 4H), 6.86 – 6.79 (m, 1H). **^{13}C NMR** (100 MHz, $CDCl_3$, 25 °C, TMS) δ (ppm): 147.33, 146.46, 145.21, 138.12, 136.72, 134.40, 134.35, 131.99, 131.03, 130.54, 130.13, 129.42, 128.96, 128.79, 128.24, 128.10, 128.05, 127.88, 127.53, 127.32, 127.03, 126.56, 126.23, 125.24, 124.76, 124.58, 124.31, 124.24, 120.57. **HR-MS** (MALDI-TOF): calculated for [$C_{47}H_{33}N_3$] [M^+] 640.2679, found; 640.2815.

Supplementary Note 3 Detailed ^{1}H NMR, ^{13}C NMR and MS data of 2-(4,5-bis(4-fluorophenyl)-1-phenyl-1H-imidazol-2-yl)-4-(di(naphthalen-2-yl)amino)phenol (**DNH-F**).

^{1}H NMR (400 MHz, $CDCl_3$, 25 °C, TMS) δ 13.41 (s, 1H), 7.75 (d, J = 7.8 Hz, 2H), 7.61 (d, J = 8.8 Hz, 2H), 7.52 (d, J = 8.0 Hz, 2H), 7.47 (dd, J = 8.7, 5.4 Hz, 2H), 7.38 (ddd, J = 15.7, 14.1, 7.2 Hz, 4H), 7.15 – 7.05 (m, 6H), 6.98 (dt, J = 10.3, 7.9 Hz, 4H), 6.84 (dd, J = 10.6, 8.1 Hz, 4H), 6.49 (t, J = 7.7 Hz, 2H), 6.38 (dd, J = 10.6, 4.8 Hz, 2H). **^{13}C NMR** (100 MHz, $CDCl_3$, 25 °C, TMS) δ (ppm) 163.81, 163.25, 155.48, 145.52, 144.63, 138.33, 136.05, 134.57, 134.46, 133.00, 132.92, 129.76, 129.25, 128.77, 128.60, 128.47, 127.65, 127.45, 126.78, 126.21, 124.30, 124.21, 123.70, 118.79, 118.70, 115.81, 115.59, 115.42, 115.21, 113.57. **HR-MS** (MALDI-TOF): calculated for [$C_{47}H_{31}F_2N_3O$] [M^{+}] 692.2441, found; 692.2568.

Supplementary Note 4 Detailed ^{1}H NMR, ^{13}C NMR and MS data of N-(3-(4,5-bis(4-fluorophenyl)-1-phenyl-1H-imidazol-2-yl)phenyl)-N-(naphthalen-2-yl)naphthalen-2-amine (**DN-F**).

^{1}H NMR (400 MHz, $CDCl_3$, 25 °C, TMS) δ (ppm): 7.76 (d, J = 7.7 Hz, 2H), 7.66 (d, J = 8.8 Hz, 2H), 7.55 (d, J = 7.8 Hz, 2H), 7.50 (dd, J = 8.7, 5.5 Hz, 2H), 7.46 (d, J = 7.8 Hz, 1H), 7.43 – 7.34 (m, 4H), 7.27 (s, 3H), 7.14 (dd, J = 8.8, 2.2 Hz, 3H), 7.01 (dd, J = 8.6, 5.4 Hz, 2H), 6.97 – 6.82 (m, 10H). **^{13}C NMR** (100 MHz, $CDCl_3$, 25 °C, TMS) δ (ppm): 163.60, 163.05, 161.12, 160.63, 147.41, 146.58, 145.16, 137.51, 136.51, 134.38, 132.79, 132.71, 131.72, 130.15, 129.58, 129.45, 128.97, 128.93, 128.84, 128.24, 127.97, 127.94, 127.52, 127.01, 126.40, 126.24, 125.05, 124.79, 124.62, 124.30, 124.04, 120.63, 115.67, 115.45, 115.17, 114.95. **HR-MS** (MALDI-TOF): calculated for [$C_{47}H_{31}F_2N_3$] [M^+] 676.2492, found; 676.2623.

Supplementary Note 5 Detailed ^{1}H NMR, ^{13}C NMR and MS data of (*E*)-3-(4-(dimethylamino)phenyl)-1-(1-hydroxynaphthalen-2-yl)prop-2-en-1-one (**CH-M**).

^{1}H NMR (400 MHz, $CDCl_3$) δ 15.24 (s, 1H), 8.49 (d, *J* = 8.4 Hz, 1H), 7.98 (d, *J* = 15.2 Hz, 1H), 7.87 (d, *J* = 9.2 Hz, 1H), 7.77 (d, *J* = 8.0 Hz, 1H), 7.64 – 7.59 (m, 3H), 7.56 – 7.51 (m, 2H), 7.29 (d, *J* = 8.8 Hz, 1H), 6.71 (d, *J* = 8.8 Hz, 2H), 3.06 (s, 6H). **^{13}C NMR** (100 MHz, $CDCl_3$) δ 193.23, 164.17, 152.43, 146.32, 137.28, 131.00, 129.89, 127.47, 125.83, 125.80, 124.54, 124.23, 122.67, 117.97, 114.78, 113.86, 111.96, 40.25. **HR-MS** (MALDI-TOF): calculated for [$C_{21}H_{19}NO_2$] [M^+] 317.1416, Found: 317.1428.

Supplementary Note 6 Detailed ^{1}H NMR, ^{13}C NMR and MS data of (*E*)-3-(4-(dimethylamino)phenyl)-1-(naphthalen-2-yl)prop-2-en-1-one (**C-M**).

^{1}H NMR (600 MHz, $CDCl_3$) δ 8.53 (s, 1H), 8.11 (dd, *J* = 8.7, 1.5 Hz, 1H), 7.99 (d, *J* = 7.8 Hz, 1H), 7.93 (d, *J* = 9 Hz, 1H), 7.90 – 7.86 (m, 2H), 7.60 – 7.54 (m, 4H), 7.50 (d, *J* = 15.6 Hz, 1H), 6.71 (d, *J* = 9 Hz, 2H), 3.05 (s, 6H). **^{13}C NMR** (150 MHz, $CDCl_3$) δ 190.56, 152.18, 145.95, 136.54, 135.36, 132.77, 130.60, 129.57, 129.52, 128.44, 128.11, 127.91, 126.71, 124.83, 122.83, 117.05, 111.96, 40.24. **HR-MS** (ESI) *m/z*: calculated for $C_{21}H_{19}NO$ $[M+H]^+$ 302.1545, Found: 302.1536.

Supplementary Note 7 Detailed ^{1}H NMR, ^{13}C NMR and MS data of (E)-3-(4-(diphenylamino)phenyl)-1-(1-hydroxynaphthalen-2-yl)prop-2-en-1-one (**CH-P**).

^{1}H NMR (400 MHz, $CDCl_3$) δ 15.03 (s, 1H), 8.50 (d, *J* = 8.0 Hz, 1H), 7.95 (d, *J* = 15.2 Hz, 1H), 7.84 (d, *J* = 8.8 Hz, 1H), 7.77 (d, *J* = 8.0 Hz, 1H), 7.65 – 7.58 (m, 2H), 7.58 – 7.52 (m, 3H), 7.34 – 7.28 (m, 5H), 7.18 – 7.11 (m, 6H), 7.05 (d, *J* = 8.8 Hz, 2H). **^{13}C NMR** (100 MHz, $CDCl_3$) δ 193.26, 164.41, 150.68, 146.88, 145.14, 137.43, 130.22, 130.15, 129.72, 127.75, 127.52, 125.99, 125.79, 125.75, 124.63, 124.48, 124.11, 121.48, 118.20, 117.60, 113.77. **HR-MS** (MALDI-TOF): calculated for [$C_{31}H_{23}NO_2$] [M^+] 441.1729, Found: 441.1753.

Supplementary Note 8 Detailed ^{1}H NMR, ^{13}C NMR and MS data of (*E*)-3-(4-(diphenylamino)phenyl)-1-(naphthalen-2-yl)prop-2-en-1-one (**C-P**).

^{1}H NMR (600 MHz, $CDCl_3$) δ 8.54 (s, 1H), 8.11 (dd, *J* = 8.4, 1.8 Hz, 1H), 7.99 (d, *J* = 8.4 Hz, 1H), 7.94 (d, *J* = 9 Hz, 1H), 7.90 (d, *J* = 8.4 Hz, 1H), 7.85 (d, *J* = 15.6 Hz, 1H), 7.62 – 7.58 (m, 2H), 7.56 – 7.54 (m, 3H), 7.33 – 7.31 (m, 4H), 7.18 – 7.17 (m, 4H), 7.14 – 7.11 (m, 2H), 7.07 (d, *J* = 8.4 Hz, 2H). **^{13}C NMR** (150 MHz, $CDCl_3$) δ 190.40, 150.31, 146.95, 144.75, 136.09, 135.47, 132.71, 129.91, 129.77, 129.64, 129.60, 128.58, 128.32, 128.02, 127.92, 126.81, 125.60, 124.70, 124.26, 121.70, 119.56. **HR-MS** (ESI) *m/z*: calculated for $C_{31}H_{23}NO$ $[M]^+$ 425.1780, Found: 425.1771.

Supplementary Note 9 Lifetime variation of ESIPT molecule depending on the excitation wavelength

Luminescence spectra of ESIPT materials as a function of the excitation wavelengths of imidazole-based molecules or charcone-based materials are not extensively investigated so far. However, similar ESIPT systems including 6,6'-dimethyl3,3'-dihydroxy-2,2'-bipyridine ($BP(OH)_2$), 2-(20-hydroxyphenyl)benzothiazole (HBT), 2-(20-hydroxyphenyl)benzoxazole (HBO), and ortho-hydroxybenzaldehyde (OHBA) are reported to identify the relationships between the excitation wavelength and emission properties of the materials[61,62]. For example, in ($BP(OH)_2$) molecule, emission from Franck-Condon excited state (the S_1 state) showed weaker fluorescence at 490 nm but the emission which are derived from higher excited states (S_2 or higher) exhibited more intense emission at 535 nm as a result of barrierless efficient ESIPT process[61]. Moreover, it was reported that the skeletal motions of the ESIPT molecules contributes to the reaction path of the proton transfer[62]. The UV-visible pump-probe spectroscopy for HBT, HBO, and OHBA with 30 femtosec resolution showed that a bending motion of the molecular skeleton reduces the proton donor-acceptor (D-A) distance, and the ESIPT process can only occur when sufficient vibrational energy is injected into the Franck-Condon state by the high-energy excitation[62]. Recent studies for the excitation wavelength and emission relationships on triazole derivatives showed a bright ESIPT luminescence upon high-energy excitation (less than 350 nm) with 16.5% PLQY, but relatively weak excimer fluorescence with 3.9% PLQY under low-energy irradiation longer than 365 nm[63]. Considering that the PLQY is proportional to the fluorescence lifetime in a system which is free from dynamic collisional quenching, high-energy excitation for our imidazole- and charcone-based molecules could induce extended fluorescence lifetime, together with more efficient ESIPT after the photoexcitation.

Supplementary Note 10 Resolution of the color-discrimination capability

The color-distinguish capability is achieved by satisfying the following three characteristics of the memristor.

Condition 1. The PSC range of each RGB color must be adequately separated to avoid any overlap, as shown in Fig. 6.

Condition 2. The PSC value difference between each state of the B color should be much larger than the highest PSC value of the states of the G and R colors. Similarly, the PSC value difference between each state of the G color should be larger than the highest PSC value of the states of the R color.

In the following Supplementary Table 5, we present an illustrative example of a 4-bit resolution RGB color-distinguishing case, based on the performance characteristics of the CH-P memristor, including its spectral responsivity and relaxation time. Firstly, the PSC range of each RGB color is adequately separated to avoid any overlap, as suggested in condition 1. Secondly, the PSC difference of each state of the blue color (7.5 mA cm^{-2}) is considerably larger than the highest PSC value of the states of the green and red colors (4.2 and 0.15 mA cm^{-2}, respectively), satisfying condition 2. Similarly, the PSC difference of each state of the green color (0.2 mA cm^{-2}) is notably larger than the highest PSC value of the state of the red color (0.15 mA cm^{-2}).

Supplementary Table 5 4-bit resolution RGB color-distinguish case of CH-P-integrated memristor.

**Blue**		
State	PSC after 50th pulse (mA cm^{-2})	Standard deviation (mA cm^{-2})
15	210.0	
14	202.5	
13	195.0	
12	187.5	
11	180.0	
10	172.5	
9	165.0	
8	157.5	$\pm 1.2 \times 10^{-2}$
7	150.0	
6	142.5	
5	135.0	
4	127.5	
3	120.0	
2	112.5	
1	105.0	
0	0	0

**Green**		
State	PSC after 50th pulse (mA cm^{-2})	Standard deviation (mA cm^{-2})
15	4.2	
14	4	
13	3.8	
12	3.6	
11	3.4	
10	3.2	
9	3	
8	2.8	$\pm 3.9 \times 10^{-4}$
7	2.6	
6	2.4	
5	2.2	
4	2.0	
3	1.8	
2	1.6	
1	1.4	
0	0	0

**Red**		
State	PSC after 50th pulse (mA cm^{-2})	Standard deviation (mA cm^{-2})
15	0.15	
14	0.14	
13	0.13	
12	0.12	
11	0.11	
10	0.10	
9	0.09	
8	0.08	$\pm 8.9 \times 10^{-6}$
7	0.07	
6	0.06	
5	0.05	
4	0.04	
3	0.03	
2	0.02	
1	0.01	
0	0	0

Under the given condition, our current synapse device can distinguish a 4-bit resolution color signal within the range of (R, G, B) = (0~15, 0~15, 0~15).

Condition 3. To ensure the aforementioned color-separation is valid, it is necessary that the deviation of the PSC value for the B color at each state should be smaller than the interval between states of the G and R colors. Additionally, the deviation of the PSC value for the G color at each state should also be smaller than the interval between states of the R color. The standard deviation values of BGR in Supplementary Table 5, as provided above, satisfy these requirements.

The deviation of the PSC value at each state for the B color, approximately 0.01 mA cm^{-2}, is comparable to the lowest PSC value of the states of the R color. Therefore, there is a possibility that distinguishing the weakest R signal could be challenging. However, it is important to note that this work serves as a proof-of-concept for the photonic synapse, showcasing its color-distinguishing characteristics even with a simple two-terminal memristor architecture. Moreover, as emphasized previously, this approach is not limited to a specific medium, making it widely applicable. We believe that by designing molecules with enhanced excited state dipole moment, improved spectral responsivity and relaxation time, we can further increase the resolution by optimizing the conductance gap between each state of each color. Consequently, this approach could serve as a platform technology for color-distinguishing synapses utilizing a simple two-terminal architecture, even with the benefit of universality and versatility.

Supplementary Note 11 Nonlinearity and symmetricity of LTP/LTD curves and weight update

To simulate the image recognition tasks of the CNN about the CIFAR-10 dataset using the NeuroSim simulator, the conductance update behaviors of the memristors are modeled by the following equations (1)-(3)[64]:

$$G_{\mathrm{LTP}} = G_{\mathrm{min}} + B\left(1 - e^{-\frac{P}{A}}\right) \tag{1}$$

$$G_{\mathrm{LTD}} = G_{\mathrm{max}} - B\left(1 - e^{-\frac{P-P_{\mathrm{max}}}{A}}\right) \tag{2}$$

$$B = \frac{G_{\mathrm{max}} - G_{\mathrm{min}}}{1 - e^{-\frac{P_{\mathrm{max}}}{A}}} \tag{3}$$

where G_{LTP} and G_{LTD} are the conductance of device for the potentiation and depression, G_{max} and G_{min} are their maximum and minimum values, P is the pulse number, and P_{max} is its maximum value that can be applied to the device to adjust its conductance. The A value is inversely proportional to nonlinearity of the LTP/LTD curves, and B is a fitting parameter, which is a function of A. The theoretical variation of the LTP/LTD curves depending on their nonlinearity values are illustrated in the following Supplementary Fig. 40a, and the LTP/LTD nonlinearity values of the DNH-F-integrated memristor, which are estimated by fitting LTP/LTD curve to the equation (1)-(3), are 1.11 and 1.05 (Supplementary Fig. 40b).

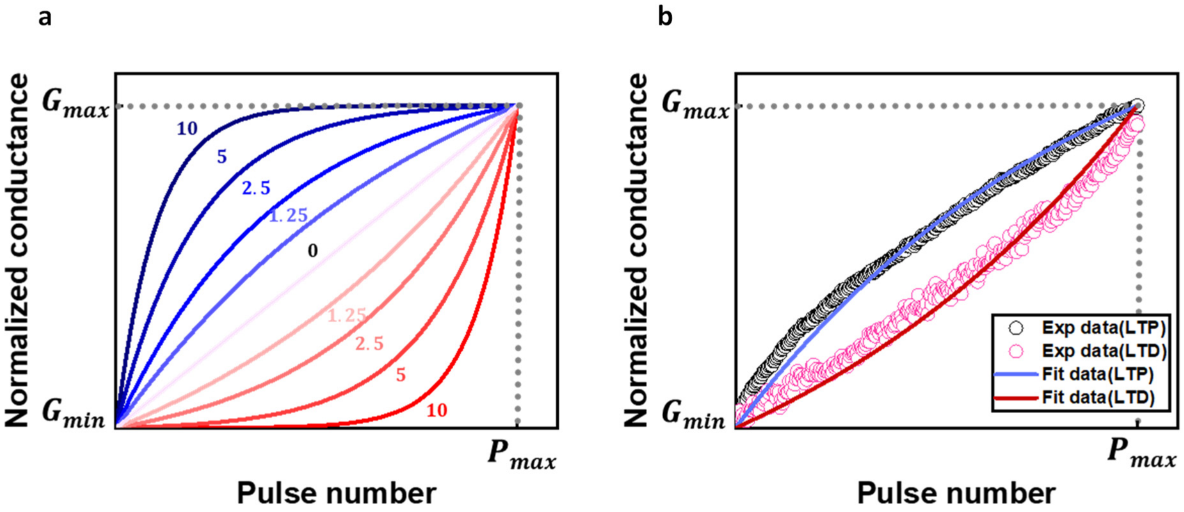

Supplementary Fig. 40 a LTP (blue) and LTD (red) curves theoretically estimated by Equations (1)-(3) with nonlinearity values between 0 and 10. **b** LTP/LTD behavior of the DNH-F-integrated memristor. The estimated nonlinearity values are 1.11 and 1.05 for LTP and LTD.

Symmetricity is defined as the reciprocal of the symmetric error[65]:

$$Symmetricity = \left(\sum_{k=1}^{k=n} \frac{\left(G_N(k) - G_N(2n-k)\right)^2}{n}\right)^{-1}$$

$$= \left(\sum_{k=1}^{k=n} \frac{([\{G(k) - G_{min}\}] - [\{G(2n-k) - G_{min}\}])^2}{n \times (G_{max} - G_{min})^2}\right)^{-1}$$

$$= \left(\sum_{k=1}^{k=n} \frac{\{G(k) - G(2n-k)\}^2}{n \times (G_{max} - G_{min})^2}\right)^{-1}$$

, where $G_N(k) = \frac{G(k) - G_{min}}{G_{max} - G_{min}}$. Supplementary Figure 41a shows $G(k)$ and $G(2n\text{-}k)$, and the perfectly symmetric (dark-cyan-colored) and asymmetric (sky-blue-colored) LTP/LTD curves, which have ∞ and 0 values, are depicted in Supplementary Fig. 41b.

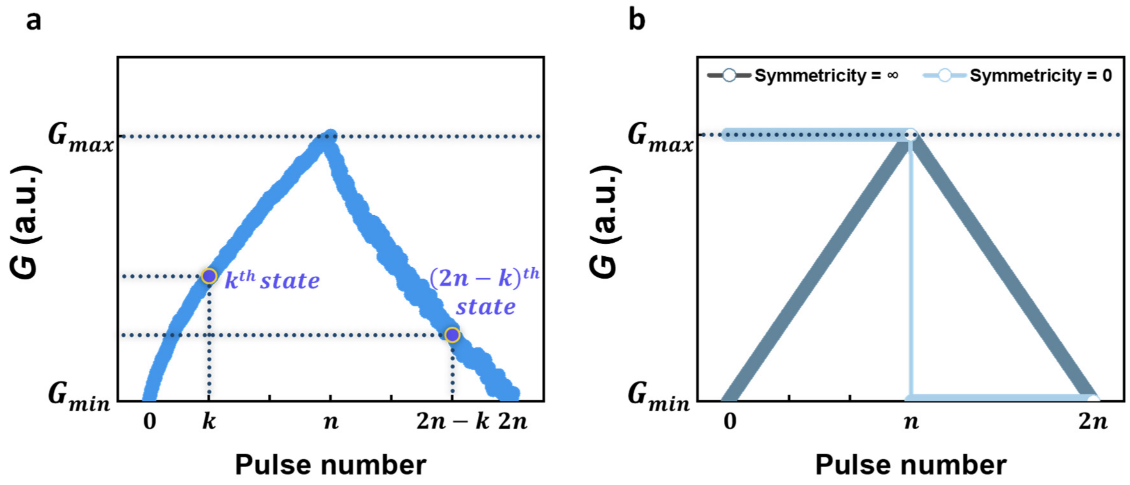

Supplementary Fig. 41 a, Conductance (G) at k^{th} and $(2n\text{-}k)^{th}$ pulses. **b**, Theoretical LTP/LTD curves with 0 and ∞ symmetricity values.

The synaptic weight (W) of each cell is defined by the conductance difference between two equivalent memristors ($W = G^+ - G^-$). In unidirectional update, the weights are increased only when G^+ increases ($W \uparrow = G^+ \uparrow - G^-$) and are decreased when G^- increases ($W \downarrow = G^+ - G^- \uparrow$). In contrast, in the bidirectional update method, since both potentiation and depression behaviors of the device participate in the weight update, the weight increase is caused by the increase of G^+ and the decrease of G^- ($W \uparrow = G^+ \uparrow - G^- \downarrow$). The weight reduction is depicted by the decrease of G^+ and the increase of G^- ($W \downarrow = G^+ \downarrow - G^- \uparrow$).

References in Supplementary Information

1. Zhou, F., Zhou, Z., Chen, J., Choy, T. H., Wang, J., Zhang, N., Lin, Z., Yu, S., Kang, J., Wong, H. -S. P. & Chai, Y. Optoelectronic resistive random access memory for neuromorphic vision sensors. *Nat. Nanotechnol.* **14**, 776-782 (2019).

2. Yang, L., Singh, M., Shen, S. -W., Chih, K. -Y., Liu, S. -W., Wu, C. -I., Chu, C. -W. & Lin, H. -W. Transparent and flexible inorganic perovskite photonic artificial synapses with dual-mode operation. *Adv. Funct. Mater.* **31**, 2008259 (2021).

3. Ma, F., Zhu, Y., Xu, Z., Liu, Y., Zheng, X., Ju, S., Li, Q., Ni, Z., Hu, H., Chai, Y., Wu, C., Kim, T. W. & Li, F. Optoelectronic perovskite synapses for neuromorphic computing. *Adv. Funct. Mater.* **30**, 1908901 (2020).

4. Zhao, L., Fan, Z., Cheng, S., Hong, L., Li, Y., Tian, G., Chen, D., Hou, Z., Qin, M., Zeng, M., Lu, X., Zhou, G., Gao, X. & Liu, J. -M. An artificial optoelectronic synapse based on a photoelectric memcapcitor. *Adv. Electron. Mater.* **6**, 1900858 (2020).

5. Wang, W., Gao, S., Li, Y., Yue, W., Kan, H., Zhang, C., Lou, Z., Wang, L. & Shen, G. Artificial optoelectronic synapses based on TiN_xO_{2-x}/MOS_2 heterojunction for neuromorphic computing and visual system. *Adv. Funct. Mater.* **31**, 2101201 (2021).

6. Gong, Y., Wang, Y., Li, R., Yang, J. -Q., Lv, Z., Xing, X., Liao, Q., Wang, J., Chen, J., Zhou, Y. & Han, S. -T. Tailoring synaptic plasticity in a perovskite QD-based asymmetric memristor. *J. Mater. Chem. C* **8**, 2985-2992 (2020).

7. Wang, Y., Gong, Y., Yang, L., Xiong, Z., Lv, Z., Xing, X., Zhou, Y., Zhang, B., Su, C., Liao, Q. & Han, S. -T. MXene-ZnO memristor for multimodal in-sensor computing. *Adv. Funct. Mater.* **31**, 2100144 (2021).

8. Zhong, W. -M., Tang, X. -G., Liu, Q. -X. & Jiang, Y. -P. Artificial optoelectronic synaptic characteristics of Bi2FeMnO6 ferroelectric memristor for neuromorphic computing. *Mater. Des*. **222**, 111046 (2022).

9. Moon, G., Min, S. Y., Han, C., Lee, S. -H., Ahn, H., Seo, S. -Y., Ding, F., Kim, S. & Jo, M. -H. Atomically thin synapse networks on van der Waals photo-memtransistors. *Adv. Mater.* **35**, 2203481 (2022).

10. Shan, X., Zhao, C., Wang, X., Wang, Z., Fu, S., Lin, Y., Zeng, T., Zhao, X., Xu, H., Zhang, X. & Liu, Y. Plasmonic optoelectronic memristor enabling fully light-modulated synaptic plasticity for neuromorphic vision. *Adv. Sci.* **9**, 2104632 (2022).

11. Sun, J., Chen, Q., Fan, F., Zhang, Z., Han, T., He, Z., Wu, Z., Yu, Z., Gao, P., Chen, D., Zhang, B. & Liu, G. A dual-mode organic memristor for coordinated visual perceptive computing. *Fundam. Res*. (2022).

12. Hao, D., Liu, D., Zhang, J., Wang, Y. & Huang, J. Lead-free perovskites-based photonic synaptic devices with logic functions. *Adv. Mater. Technol.* **6**, 2100678 (2021).

13. Shrivastava, S., Lin, Y. -T., Pattanayak, B., Pratik, S., Hsu, C. -C., Kumar, D., Lin, A. S. & Tseng, T. -Y. Zn_2SnO_4 thin film based nonvolatile positive optoelectronic memory for neuromorphic computing. *ACS Appl. Electron. Mater.* **4**, 1784-1793 (2022).

14. Zhao, P., Ji, R., Lao, J., Xu, W., Jiang, C., Luo, C., Lin, H., Peng, H. & Duan, C. -G. Two-terminal organic optoelectronic synapse based on poly(3-hexylthiophene) for neuromorphic computing. *Org. Electron.* **100**, 106390 (2022).

15. Zhu, Y., Wu, C., Xu, Z., Liu, Y., Hu, H., Guo, T., Kim, T. W., Chai, Y. & Li, F. Light-emitting memristors for optoelectronic artificial efferent nerve. *Nano Lett.* **21**, 6087-6094 (2021).

16. Li, C., Ilyas, N., Wang, J., Li, Y., Luo, H., Li, D., Gu, D., Liu, F., Jiang, Y. & Li, W. Nanostructured $CuAlO_2$@ZnO optoelectronic device for artificial synaptic applications. *Appl. Surf. Sci.* **611**, 155682

(2023).

17. Zhao, P., Ji, R., Lao, J., Jiang, C., Tian, B., Luo, C., Lin, H., Peng, H. & Duan, C. -G. Multifunctional two-terminal optoelectronic synapse based on zinc oxide/poly(3-hexylthiophene) heterojunction for neuromorphic computing. *ACS Appl. Polym. Mater.* **4**, 5688-5695 (2022).

18. Akbari, M. K. & Zhuiykov, S. A bioinspired optoelectronically engineered artificial neurorobotics device with sensorimotor functionalities. *Nat. Commun.* **10**, 3873 (2019).

19. Xing, J., Zhao, C., Zou, Y., Kong, W., Yu, Z., Shan, Y., Dong, Q., Zhou, D., Yu, W. & Guo, C. Modulating the optical and electrical properties of $MAPbBr_3$ single crystals via voltage regulation engineering and application in memristors. *Light Sci. Appl.* **9**, 111 (2020).

20. Seo, S., Lee, J. -J., Lee, R. -G., Kim, T. H., Park, S., Jung, S., Lee, H. -K., Andreev, M., Lee, K. -B., Jung, K. -S., Oh, S., Lee, H. -J., Kim, K. S., Yeom, G. Y., Kim, Y. -H. & Park, J. -H. An optogenetics-inspired flexible van der waals optoelectronic synapse and its application to a convolutional neural network. *Adv. Mater.* **33**, 2102980 (2021).

21. Duan, J., Chava, P., Ghorbani-Asl, M., Erb, D., Hu, L., Krasheninnikov, A. V., Schneider, H., Rebohle, L., Erbe, A., Helm, M., Zeng, Y. -J., Zhou, S. & Prucnal, S. Enhanced trion emission in monolayer $MoSe_2$ by constructing a type-1 van der waals heterostructure. *Adv. Mater.* **33**, 2104960 (2021).

22. Zhao, Y., Yu, D., Liu, Z., Li, S. & He, Z. Memtransistors based on non-layered In_2S_3 two-dimensional thin films with optical-modulated multilevel resistance states and gate-tunable artificial synaptic plasticity. *IEEE Access* **8**, 106726-106734 (2020).

23. Liu, Y., Wu, L., Liu, Q., Liu, L., Ke, S., Peng, Z., Shi, T., Yuan, X., Huang, H., Li, J., Ye, C., Chu, P. K., Wang, J. & Yu, X. -F. Topochemical synthesis of copper phosphide nanoribbons for flexible optoelectronic memristors. *Adv. Funct. Mater.* **32**, 2110900 (2022).

24. Li, Y., Ji, R., Zhao, P., Lao, J., Jiang, C., Tian, B., Luo, C., Lin, H., Peng, H. & Duan, C. -G. Transparent optoelectronic synapse based on a CuI electrode for arithmetic operation. *ACS Appl. Electron. Mater.* **4**, 1989-1996 (2022).

25. Hu, L., Yang, J., Wang, J., Cheng, P., Chua, L. O. & Zhuge, F. All-optically controlled memristor for optoelectronic neuromorphic computing. *Adv. Funct. Mater.* **31**, 2005582 (2021).

26. Liu, L., Cheng, Z., Jiang, B., Liu, Y., Zhang, Y., Yang, F., Wang, J., Yu, X. -F., Chu, P. K. & Ye, C. Optoelectronic artificial synapses based on two-dimensional transitional-metal trichalcogenide. *ACS Appl. Mater. Interfaces* **13**, 30797-30805 (2021).

27. Hou, Y. -X., Li, Y., Zhang, Z. -C., Li, J. -Q., Qi, D. -H., Chen, X. -D., Wang, J. -J., Yao, B. -W., Yu, M. -X., Lu, T. -B. & Zhang, J. Large-scale and flexible optical synapses for neuromorphic computing and integrated visible information sensing memory processing. *ACS Nano* **15**, 1497-1508 (2021).

28. Gao, S., Liu, G., Yang, H., Hu, C., Chen, Q., Gong, G., Xue, X., Yi, X., Shang, J. & Li, R. -W. An oxide schottky junction artificial optoelectronic synapse. *ACS Nano* **13**, 2634-2642 (2019).

29. Xu, H., Akbari, M. K., Wang, S., Chen, S., Kats, E., Verpoort, F., Hu, J. & Zhuiykov, S. Tunability of near infrared opto-synaptic properties of thin MoO_3 films fabricated by atomic layer deposition. *Appl. Surf. Sci.* **593**, 153399 (2022).

30. Chen, H., Lv, L., Wei, Y., Liu, T., Wang, S., Shi, Q. & Huang, H. Self-powered flexible artificial synapse for near-infrared light detection. *Cell Rep. Phys. Sci.* **2**, 100507 (2021).

31. Wang, T. -Y., Meng, J. -L., Li, Q. -X., He, Z. -Y., Zhu, H., Ji, L., Sun, Q. -Q., Chen, L. & Zhang, D. W.

Reconfigurable optoelectronic memristor for in-sensor computing applications. *Nano Energy* **89**, 106291 (2021).

32. Pei, Y., Yan, L., Wu, Z., Lu, J., Zhao, J., Chen, J., Liu, Q. & Yan, X. Artificial visual perception nervous system based on low-dimensional material photoelectric memristors. *ACS Nano* **15**, 17319-17326 (2021).

33. Park, H. -L., Kim, H., Lim, D., Zhou, H., Kim, Y. -H., Lee, Y., Park, S. & Lee, T. -W. Retina-inspired carbon nitride-based photonic synapses for selective detection of UV light. *Adv. Mater.* **32**, 1906899 (2020).

34. Lv, Z., Chen, M., Qian, F., Roy, V. A. L., Ye, W., She, D., Wang, Y., Xu, Z. -X., Zhou, Y. & Han, S. -T. Mimicking neuroplasticity in a hybrid biopolymer transistor by dual modes modulation. *Adv. Funct. Mater.* **29**, 1902374 (2019).

35. Yang, C., Qian, J., Jiang, S., Wang, H., Wang, Q., Wan, Q., Chan, P. K. L., Shi, Y. & Li, Y. An optically modulated organic schottky-barrier planar-diode-based artificial synapse. *Adv. Opt. Mater.* **8**, 2000153 (2020).

36. Alquraishi, W., Sun, J., Qiu, W., Liu, W., Huang, Y., Jin, C. & Gao, Y. Mimicking optoelectronic synaptic functions in solution-processed In-Ga-Zn-O phototransistors. *Appl. Phys. A* **126**, 431 (2020).

37. Qian, C., Oh, S., Choi, Y., Kim, J. -H., Sun, J., Huang, H., Yang, J., Gao, Y., Park, J. -H. & Cho, J. H. Solar-stimulated optoelectronic synapse based on organic heterojunction with linearly potentiated synaptic weight for neuromorphic computing. *Nano Energy* **66**, 104095 (2019).

38. Shi, Q., Liu, D., Hao, D., Zhang, J., Tian, L., Xiong, L. & Huang, J. Printable, ultralow-power ternary synaptic transistors for multifunctional information processing system. *Nano Energy* **87**, 106197 (2021).

39. Lee, M., Nam, S., Cho, B., Kwon, O., Lee, H. U., Hahm, M. G., Kim, U. J. & Son, H. Accelerated learning in wide-band-gap AlN artificial photonic synaptic devices: impact on suppressed shallow trap level. *Nano Lett.* **21**, 7879-7886 (2021).

40. Ahmad, T., Kuriakose, S., Mayes, E. L. H., Ramanathan, R., Bansal, V., Bhaskaran, M., Sriram, S. & Walia, S. Optically stimulated artificial synapse based on layered black phosphorus. *Small* **15**, 1900966 (2019).

41. Mao, J. -Y., Hu, L., Zhang, S. -R., Ren, Y., Yang, J. -Q., Zhou, L., Zeng, Y. -J., Zhou, Y. & Han, S. -T. Artificial synapses emulated through a light mediated organic-inorganic hybrid transistor. *J. Mater. Chem. C* **7**, 48-59 (2019).

42. Mburu, M. M., Lu, K. -T., Prine, N. L., Au-Duong, A. -N., Chiang, W. -H., Gu, X. & Chiu, Y. -C. Conjugated polymer-wrapped single-wall carbon nanotubes for high-mobility photonic/electrical fully modulated synaptic transistor. *Adv. Mater. Technol.* **7**, 2101506 (2022).

43. Zhang, Q., Ye, X., Zheng, Y., Wang, Y., Li, L., Gao, Z., Wu, J., Dong, H., Geng, D. & Hu, W. Controllable growth of centimeter-scale 2D crystalline conjugated polymers for photonic synaptic transistors. *J. Mater. Chem. C* **10**, 2681-2689 (2022).

44. Pradhan, B., Das, S., Li, J., Chowdhury, F., Cherusseri, J., Pandey, D., Dev, D., Krishnaprasad, A., Barrios, E., Towers, A., Gesquiere, A., Tetard, L., Roy, T. & Thomas, J. Ultrasensitive and ultrathin phototransistors and photonic synapses using perovskite quantum dots grown from graphene lattice. *Sci. Adv.* **6**, eaay5225 (2020).

45. Wang, R., Chen, P., Hao, D., Zhang, J., Shi, q., Liu, D., Li, L., Xiong, L., Zhou, J. & Huang, J. Artificial synapses based on lead-free perovskite floating-gate organic field-effect transistors for supervised and unsupervised learning. *ACS Appl. Mater. Interfaces* **13**, 43144-43154 (2021).

46. Elseman, A. M., Shalan, A. E., Sajid, S., Rashad, M. M., Hassan, A. M. & Li, M. Copper-substituted lead

perovskite materials constructed with different halides for working $(CH_3NH_3)_2CuX_4$-Based perovskite solar cells from experimental and theoretical view. *ACS Appl. Mater. Interfaces* **14**, 11699-11707 (2022).

47. Li, X., Yu, B., Wang, B., Bi, R., Li, H., Tu, K., Chen, G., Li, Z., Huang, R. & Li, M. Complementary photo-synapses based on light-stimulated porphyrin-coated silicon nanowires field-effect transistors. *Small* **17**, 2101434 (2021).

48. Periyal, S. S., Jagadeeswararao, M., Ng, S. E., John, R. A. & Mathews, N. Halide perovskite quantum dots photosensitized-amorphous oxide transistors for multimodal synapses. *Adv. Mater. Technol.* **5**, 2000514 (2020).

49. Islam, M. M., Dev, D., Krishnaprasad, A., Tetard, L. & Roy, T. Optoelectronic synapse using monolayer MoS_2 field effect transistors. *Sci. Rep.* **10**, 21870 (2020).

50. Zhang, J., Sun, T., Zeng, S., Hao, D., Yang, B., Dai, S., Liu, D., Xiong, L., Zhao, C. & Huang, J. Tailoring neuroplasticity in flexible perovskite QDs-based optoelectronic synaptic transistors by dual modes modulation. *Nano Energy* **95**, 106987 (2022).

51. Xu, M., Xu, T., Yu, A., Wang, H., Wang, H., Zubair, M., Luo, M., Shan, C., Guo, X., Wang, F., Hu, W. & Zhu, Y. Optoelectronic synapses based on photo-induced doping in MoS_2/h-BN field-effect transistors. *Adv. Opt. Mater.* **9**, 2100937 (2021).

52. Cho, S. S., Kim, J., Jeong, S., Kwon, S. M., Jo, C., Kwak, J. Y., Kim, D. H., Cho, S. W., Kim, Y. -H. & Park, S. K. Highly adaptive and energy efficient neuromorphic computation enabled by deep-spike heterostructure photonic neuro-transistors. *Nano Energy* **104**, 107991 (2022).

53. Yin, L., Huang, W., Xiao, R., Peng, W., Zhu, Y., Zhang, Y., Pi, X. & Yang, D. Optically stimulated synaptic devices based on the hybrid structure of silicon nanomembrane and perovskite. *Nano Lett.* **20**, 3378−3387 (2020).

54. Ou, Q., Yang, B., Zhang, J., Liu, D., Chen, T., Wang, X., Hao, D., Lu, Y. & Huang, J. Degradable photonic synaptic transistors based on natural biomaterials and carbon nanotubes. *Small* **17**, 2007241 (2021).

55. Li, X., Li, S., Tang, B., Liao, J. & Chen, Q. A vis-SWIR photonic synapse with low power consumption based on WSe_2/In_2Se_3 ferroelectric heterostructure. *Adv. Electron. Mater.* **8**, 2200343 (2022).

56. Ercan, E., Lin, Y. -C., Yang, W. -C. & Chen, W. -C. Self-assembled nanostructures of quantum dot/conjugated polymer hybrids for photonic synaptic transistors with ultralow energy consumption and zero-gate bias. *Adv. Funct. Mater.* **32**, 2107925 (2022).

57. Chen, T., Wang, X., Hao, D., Dai, S., Ou, Q., Zhang, J. & Huang, J. Photonic synapses with ultra-low energy consumption based on vertical organic field-effect transistors. *Adv. Opt. Mater.* **9**, 2002030 (2021).

58. Si, M., Wang, D., Zhao, R., Pan, D., Zhang, C., Yu, C., Lu, X., Zhao, H. & Bai, Y. Local electric-field-driven fast Li diffusion kinetics at the piezoelectric $LiTaO_3$ modified Li-rich cathode-electrolyte interphase. *Adv. Funct. Mater.* **29**, 1902538 (2019).

59. Hou, X., Liu, C., Ding, Y., Liu, L., Wang, S. & Zhou, P. A logic-memory transistor with the integration of visible information sensing-memory-processing. *Adv. Sci.* **7**, 2002072 (2020).

60. Kim, M. -K. & Lee, J. -S. Synergistic improvement of long-term plasticity in photonic synapses using ferroelectric polarization in hafnia-based oxide-semiconductor transistors. *Adv. Mater.* **32**, 1907826 (2020).

61. Ulrich, G., Natasi, F., Retailleau, P., Puntoriero, F., Ziessel, R. & Campagna, S. Luminescent excited-state intramolecular proton-transfer (ESIPT) dyes based on 4-alkyne-functionalized [2,2'-bipyridine]-3,3'-diol dyes. *Chem. Eur. J.* **14**, 4381–4392 (2008).

62. Lochbrunner, S., Stock, K. & Riedle, E. Direct observation of the nuclear motion during ultrafast intramolecular proton transfer. *J. Mol. Struct.* **700**, 13–18 (2004).

63. Zhang, Y., Yang, H., Ma, H., Bian, G., Zang, Q., Sun, J., Zhang, C., An, Z. & Wong, W. -Y. Excitation wavelength dependent fluorescence of an ESIPT triazole derivative for amine sensing and anti-counterfeiting applications. *Angew. Chem. Int. Ed.* **58**, 8773-8778 (2019).

64. Chen, P. -Y., Peng, X. & Yu, S. NeuroSim: A circuit-level macro model for benchmarking neuro-inspired architectures in online learning. *IEEE Trans. Comput.-Aided Des. Integr. Circuits Syst.* **37**, 3067-3080 (2018).

65. Kim, D., Bang, H., Baac, H. W., Lee, J., Truong, P. L., Jeong, B. H., Appadurai, T., Park, K. K., Heo, D., Nam, V. B., Yoo, H., Kim, K., Lee, D., Ko, J. H. & Park, H. J. Room-temperature-processable highly reliable resistive switching memory with reconfigurability for neuromorphic computing and ultrasonic tissue classification. *Adv. Funct. Mater.* **33**, 2213064 (2023).
